# XAFS and DFT Insights into the Kinetics and Mechanisms of Technetium Reduction by Nanoparticulate Magnetite

**DOI:** 10.1021/acs.est.6c01654

**Published:** 2026-06-23

**Authors:** Thomas Zimmermann, Natalia Mayordomo, Augusto F. Oliveira, Felix Brandt, Martina Klinkenberg, Juri Barthel, Dieter Schild, Kerstin Hockmann, Thorsten Stumpf, Andreas C. Scheinost

**Affiliations:** † Institute of Resource Ecology, 28414 Helmholtz-Zentrum Dresden-Rossendorf, Bautzner Landstraße 400, 01328 Dresden, Germany; ‡ The Rossendorf Beamline at ESRF—The European Synchrotron, CS40220, 38043 Grenoble Cedex 9, France; § Institute of Fusion Energy and Nuclear Waste Management (IFN-2), Forschungszentrum Jülich GmbH, 52428 Jülich, Germany; ∥ Institute for Nuclear Waste Disposal, Karlsruhe Institute of Technology, P.O. Box 3640, D-76021 Karlsruhe, Germany; ° Ernst Ruska-Centre (ER-C 2), Forschungszentrum Jülich GmbH, 52425 Jülich, Germany; ⊥ Applied Geochemistry, Institute of Earth and Environmental Sciences, University of Freiburg, 79104 Freiburg im Breisgau, Germany

**Keywords:** iron oxide, maghemite, Tc, incorporation, substitution, nanoparticles, pollutant

## Abstract

Radioactive technetium-99 (^99^Tc) is present in nuclear and medical wastes. Its immobilization by magnetite (Fe^II^Fe^III^_2_O_4_) has been studied in the last decades, showing that magnetite reduces pertechnetate (Tc^VII^O_4_^–^) to Tc^IV^, which is either incorporated into the magnetite structure or forms Tc^IV^–Tc^IV^-dimers attached to the magnetite surface. The distribution between both phases and the incorporation mechanism remain, however, unclear. Therefore, we investigated the molecular environment of Tc after contacting Tc^VII^ with synthesized nanoparticulate magnetite as a function of pH (2–13) and time (up to 7 weeks). X-ray absorption spectroscopy was combined with density functional theory (DFT) simulations to decipher the mechanism of Tc^IV^ incorporation. We observed that the sorption of Tc^IV^–Tc^IV^-dimers initially occurs at pH 5 and pH 7, while Tc^IV^ incorporation in magnetite prevails at longer times and at pH 10. We suggest that Tc^IV^–Tc^IV^-dimer sorption on magnetite is due to (surficial) maghemitization of the magnetite nanoparticles, whereas Tc^IV^ incorporation is due to the electron transfer from sorbed Fe^2+^ through magnetite and subsequent release of Fe^II^ in solution (redox conveyor belt model), “burying” Tc^IV^ into the magnetite structure. DFT calculations indicate that Tc^IV^ incorporates in magnetite by an exchange of two Fe^II^ atoms for one Tc^IV^, keeping the charge balanced by creating a vacancy.

## Graphical abstract

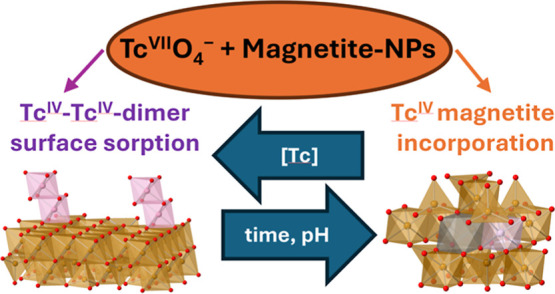

## Introduction

Technetium-99 (^99^Tc) is a beta-emitter with a long half-life (*t*_1/2_ ≈ 2.1 × 10^5^ a) and is present in significant quantities in spent nuclear fuel due to its high fission yields (∼6% for fission of ^235^U and ^239^Pu).^1^ ^99^Tc is also created in medicinal waste, since it is the daughter nuclide of the metastable technetium-99 (^99m^Tc), which is the most commonly used isotope in radiodiagnosis.^2^ Tc can occur across a wide range of oxidation states (−I to +VII),^3^ with +VII and +IV being the most common oxidation states under environmental conditions.^4^ Tc^VII^ prevails as pertechnetate anion (Tc^VII^O_4_^–^), which barely interacts with mineral surfaces, resulting in a high Tc mobility in aquifers.^5^ Tc^IV^ is less mobile since it is more reactive and forms low-soluble precipitates (e.g., Tc^IV^O_2_·*x*H_2_O) in aqueous media.^6,7^ Fe^II^-bearing minerals such as magnetite,^8^ pyrite,^9^ Fe­(OH)_2_,^10^ or chukanovite^11^ have been shown to immobilize Tc due to the reduction of Tc^VII^O_4_^–^ to Tc^IV^. The identified Tc^IV^ species of Fe^II^-driven redox processes were not only Tc^IV^O_2_·*x*H_2_O polymers but also Tc^IV^ sorption on and incorporation into (secondary) mineral phases.^8–11^

Magnetite (Fe^II^Fe^III^_2_O_4_) is a mineral that is ubiquitous in the environment under slightly reducing conditions and is an anaerobic corrosion product of steel; thus, its occurrence is expected in nuclear waste repositories.^12^ This has motivated several studies on the interaction of Tc with magnetite.^8,10,13–20^ Magnetite has tetrahedral sites that are occupied by Fe^III^ (Fe_tetr_), while the octahedral sites are occupied by an equal amount of Fe^II^ and Fe^III^ (Fe_oct_).^21^ This ideal composition is commonly referred to as stoichiometric magnetite, whereas deviations from this ideal Fe^II^/Fe^III^ distribution are described as nonstoichiometric magnetite. The electrons of octahedrally coordinated Fe are not localized, and their charge is hence considered as +2.5 above the Verwey transition temperature (>120 K).^22^

Previous studies were carried out by Lukens et al.^17,23^ on the coprecipitation method to synthesize different iron spinel phases with structurally incorporated Tc by adding Tc^VII^O_4_^–^ during the synthesis procedure. Marshall et al.^15^ investigated specifically magnetite at high pH with mineral transformation from ferrihydrite to magnetite occurring in the presence of Tc^VII^O_4_^–^. They reported that technetium was reduced to Tc^IV^ and replaces octahedral Fe^III^, leading to the creation of vacancies to balance the electric charges. This hypothesis was supported by an identical ionic radius and thereby structural interchangeability of Fe^III^ and Tc^IV^ (*r* = 78.5 pm for crystal radii^24^). In a more recent review article, this interpretation was revisited, and it was argued that Tc^IV^ is more likely to substitute for octahedral Fe^II^ rather than Fe^III^ in magnetite.^25^ Extended X-ray absorption fine-structure (EXAFS) corroborated the octahedral coordination of Tc^IV^. Since the formation of vacancies has been previously observed during the oxidation of magnetite to maghemite, a process called maghemitization,^26^ this is a likely mechanism for charge balance. Tc reoxidation experiments carried out in air^17,23^ measured only small amounts of Tc^VII^O_4_^–^ in solution (<10% for magnetite and titanomagnetite phases), demonstrating that Tc was shielded from reoxidation and suggesting that Tc^IV^ incorporation was the predominant mechanism. Similar Tc^IV^ reoxidation results have been obtained by Marshall et al.^15^ and Um et al.^16^

Kobayashi et al.^20^ investigated the interaction of Tc^VII^ with presynthesized nanoparticulate (NP) magnetite at pH 6.0 and pH 7.5 after two months of equilibration. They found the complete incorporation of Tc^IV^ in the magnetite structure. Formation of a surface precipitate was discussed but deemed unlikely due to the fit of high Fe coordination numbers using EXAFS. Building up on these studies, Yalcintas et al.^8^ aimed at studying the additional possible contribution of Tc^IV^-sorbed species on magnetite by modifying the Tc loadings and solid/liquid ratios in magnetite at pH 9.0 and 6 weeks of equilibration. In all of the investigated samples, Tc^VII^ was fully reduced to Tc^IV^. They identified Tc^IV^ incorporation in magnetite and the formation of Tc^IV^–Tc^IV^ dimers sorbed on magnetite. The authors hypothesized that the formation of sorbed Tc^IV^–Tc^IV^-dimers was connected with lower magnetite solubility at pH 9.0 and suggested that the incorporation of Tc^IV^ in magnetite was higher at pH 6.5 due to the then higher solubility of magnetite and a possible recrystallization of magnetite. These works^8,20^ suggested hence that the Tc retention mechanism by magnetite is dependent on the pH and magnetite solubility.

All studies using EXAFS^8,10,15–17,20,23^ found Tc–Fe shells at ∼3.1 and ∼3.5 Å. While the distance of 3.5 Å is very similar to Fe_oct_–Fe_tetr_ in magnetite (3.48 Å),^27^ the distance of 3.1 Å (possibly Tc–Fe_oct_) is significantly different from the 2.97 Å for Fe_oct_–Fe_oct_ in magnetite.^27^ This change of atomic distance induces a structural transformation of the crystal lattice when incorporation of Tc^IV^ occurs. It is important to highlight that in the cited papers, it has been assumed that Tc^IV^ incorporation proceeds through the substitution of Fe^III^ by Tc^IV^. If this were the case, no significant structural changes in magnetite should occur as Fe^III^ and Tc^IV^ present the same ionic radius.^24^

The existing literature dealing with Tc and presynthesized NP-magnetite^8,15,20^ covers only relatively narrow pH ranges (only high pH (10.5–13.1),^15^ pH 9.0,^8^ pH 6.0–7.0 ^20^). Furthermore, EXAFS spectroscopy as a state-of-the-art method to elucidate the molecular environment of Tc in Tc-loaded iron minerals is a tedious task, as the coupled Fe/Tc redox reaction may lead to several new phases, which are difficult to distinguish by EXAFS spectroscopy alone, even when advanced chemometrics have been employed.^8,11^ This is first due to the fact that the secondary iron minerals present very similar radial distribution functions, which makes them hard to differentiate by EXAFS.

Second, Tc^IV^ could incorporate into these minerals, form surface complexes, and/or precipitate as separate Tc^IV^O_2_·*x*H_2_O. Thus, a clear identification of the Tc environment is rather difficult since Tc^IV^ could interact with various Fe minerals by different mechanisms.^9,28^ To solve this problem, several Fe minerals, including magnetite, goethite,^16,29^ and hematite,^17,30^ have been used as references to fit the Tc^IV^ EXAFS data, and advanced analysis methods including iterative transformation factor analysis (ITFA),^31^ self-organizing maps,^11^ or Monte Carlo target transformation factor analysis based on Raman experiments have been applied.^28^

In the current work, our goal was to systematically investigate Tc^VII^ reaction with NP-magnetite by varying pH (2–13) and reaction time (15 min to 7 weeks) in order to clarify the factors affecting Tc immobilization mechanisms by magnetite in general and specifically the distribution between Tc^IV^–Tc^IV^-dimers sorbed on magnetite and Tc^IV^ incorporation in magnetite. State-of-the-art methods were used to investigate mineralogical changes: powder X-ray diffraction (pXRD), transmission electron microscopy (TEM), and X-ray photoelectron spectroscopy (XPS). X-ray absorption spectroscopy (XAS) was used to identify and calculate the Tc species distribution in magnetite samples, and finally, density functional theory (DFT) was applied to confirm the XAS-derived local structure and to elucidate the charge compensation mechanisms required for Tc^IV^ incorporation in the magnetite lattice.

## Experimental Section

All experiments were carried out in a glovebox in a N_2_ atmosphere (Glovebox-System GS050912). The oxygen content was always below 2 ppm. ^99^Tc is a radioactive isotope with weak β^–^-emission and has to be handled in an authorized laboratory with regulated handling of radioactive material. The Milli-Q water (resistivity of 18.2 MΩ·cm, Water Purified) used for the experiments was boiled for at least 2 h for degassing, sealed, and cooled down to room temperature before its use inside the glovebox.

### Magnetite Synthesis

NP-magnetite was synthesized following a previous procedure.^32^ First, 15.9 g of Fe^II^Cl_2_·4 H_2_O and 43.2 g of Fe^III^Cl_3_·6H_2_O were independently dissolved in 88.5 mL of 0.1 M HCl and consecutively mixed, yielding 200 mL of a 0.4 M Fe^II^ and 0.8 M Fe^III^ solution. For Tc-magnetite coprecipitation (CoPrec) samples used as references, also a 10 mM Tc^VII^O_4_^–^ stock was added to the Fe solution (Table S1). Subsequently, 250 mL of 6 M NH_4_OH was rapidly added while shaking intensively by hand. The immediately formed black precipitate was distributed in 50 mL of Greiner tubes and washed thrice by adding 20 mL of Milli-Q H_2_O, followed by centrifugation (6000 rpm for 10 min) and removal of the supernatant. The NP-magnetite was characterized for purity and particle size by pXRD (Figure S1) and TEM (Figure S2), with TEM showing a rather uniform particle size of 13 ± 3 nm before and no significant change after the sorption of Tc.

### Tc Batch Sorption Experiments

An aqueous 10 mM KTc^VII^O_4_ stock solution was used for the experiments. Magnetite suspensions (4 g/L) were equilibrated at given pH values 1 day prior to Tc addition (pH_ini_). The pH adjustment was performed by adding aliquots of NaOH or HCl of concentrations ranging from 0.01 to 1.0 M. After Tc addition, the pH was regularly checked and readjusted (Section S3). The resulting suspensions were shaken in a horizontal shaker for a given time, after which pH (pH_end_) and *Eh* values were measured (Section S3). Samples were centrifuged (6000 rpm for 10 min; 600 g). Subsequently, the supernatant was analyzed for Tc and Fe concentrations, using the Ferrozine assay for differentiation of Fe^2+^/Fe^3+^ (Section S3).^33^ The solid phase was characterized via TEM, XAS, and XPS (see Sections S4, S5, and S7, respectively). Tc sorption experiments were performed to evaluate pH effects, with initial pH (pH_ini_) in the range of 2.0–13.0 in steps of full units, a nominal Tc loading of 600 ppm, i.e., 24.3 μM Tc^VII^ in 50 mL of H_2_O containing 200 mg of magnetite, and a reaction time of 4 weeks (Table S2). To evaluate process kinetics, we investigated Tc sorption after 1, 14, 28, and 49 days, each at three different pH_ini_ (5.0, 7.0, 10.0) (Table S3). Additionally, kinetics of Tc removal were investigated from 15 min up to a few hours (Table S4).

## Results and Discussion

### Tc Sorption by NP-Magnetite

Figure 1 shows the results of the batch sorption experiments from pH 2.0 to 13.0 after 4 weeks of equilibration of Tc-magnetite suspensions. While Tc removal (blue squares in Figure 1) was close to zero for pH < 3.0, it increased to 98% for pH > 5.0 (no value could be obtained for pH 4.0 due to a strong pH drift of this sample). The *Eh* value continually decreased with pH (red circles in Figure 1), intersecting at about pH 3.5 with the change from Tc^VII^ to the Tc^IV^ stability field and coinciding with an increase of Tc retention, suggesting that the steep rise in Tc removal between pH 3.0 and 5.0 is due to the reduction of Tc^VII^O_4_^–^ to Tc^IV^. Note also the release of Fe^2+^ starting below pH 6.5 and increasing with decreasing pH (light green up triangles). Below pH 5.5, also Fe^3+^ is released, indicating the onset of a more stoichiometric magnetite dissolution as discussed below.

**Figure 1:**
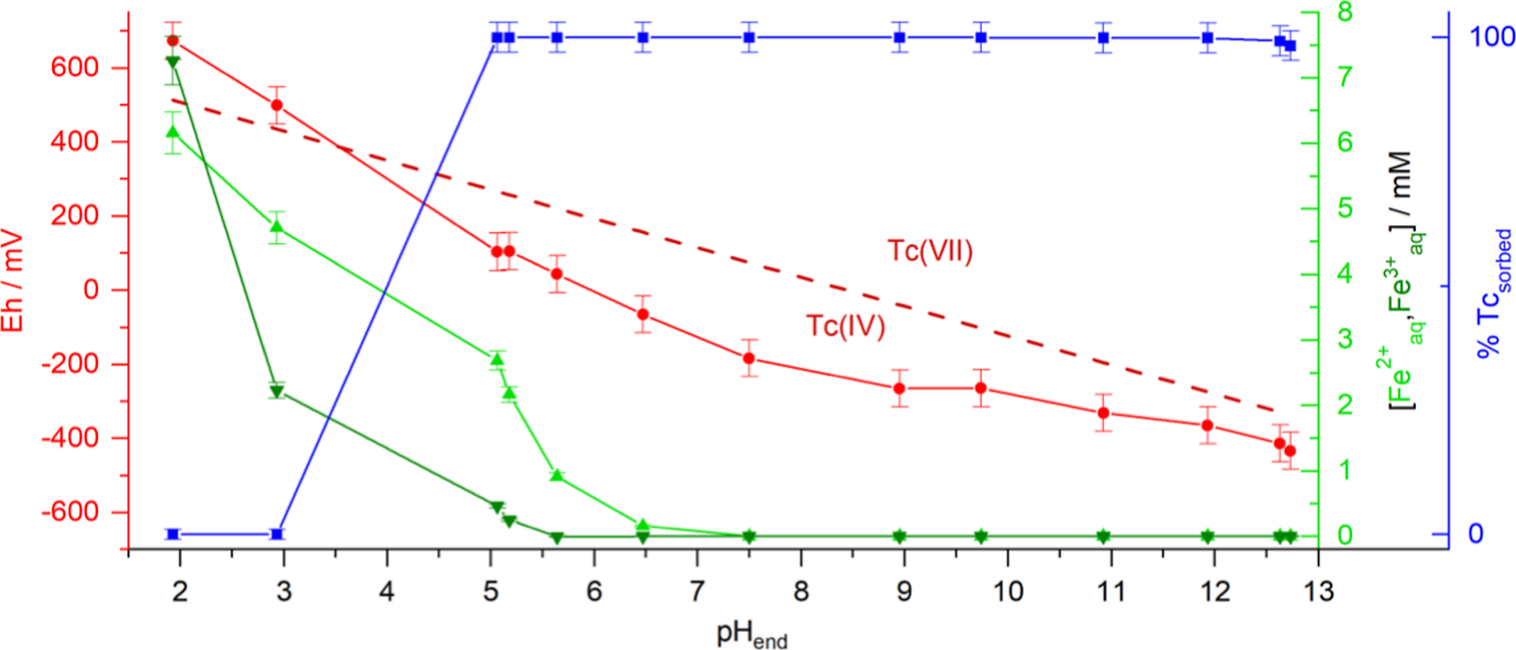
Tc^VII^O_4_^–^ removal by NP-magnetite (% Tc sorbed, blue squares), redox potential (*Eh*, red circles), [Fe^2+^]_aq_ (light green, upward facing triangles), and [Fe^3+^]_aq_ (dark green, downward facing triangles) as a function of pH (after 4 weeks of equilibration). The dashed line (dark red) divides the stability fields for Tc^VII^ above and Tc^IV^ below, calculated with the values from ref 34.

The kinetics of Tc retention by magnetite was investigated at pH 5.0, 7.0, and 10.0 (Table S4). After 2 h, Tc was at all pH values below detection limit (∼10^–8^ M) of liquid scintillation counting, indicating a Tc removal from solution of >99%. While this removal is very fast, the subsequent structural changes likely occur on longer time scales. STEM–EDX was used to investigate the particle morphology and localization of Tc on coprecipitates with 2 and 5 wt % Tc (Section S4). Particle morphology and size did not change significantly during the experiment. The elemental mappings show a general correlation between the Tc and the Fe distribution (see Figure S3), consistent with the XAS results. Unfortunately, the very low Tc signal did not allow us to conclude whether Tc is concentrated on the surfaces of the particles or is evenly distributed. However, none of the micrographs indicates the presence of separate Tc-phases larger than a few nanometers.

### Oxidation State and Local Structure of Solid-Associated Tc Species

Tc K-edge XAS spectra were collected at the Rossendorf Beamline (BM20) at the European Synchrotron Radiation Facility in Grenoble, France.^35^ Tables S1–S4 list the 12 sorption samples sorted by pH (5.0, 7.0, 10.0) and time (1, 14, 28, and 49 days) (Table S3), and the six coprecipitation samples synthesized as references (CoPrec, Table S1), which are labeled according to the amount of Tc doping (0.6, 300, 10, 20, 30, 50 kppm). The Tc K-edge XANES spectra (Figure S4A) confirm that Tc is present in all the samples in its tetravalent oxidation state, indicating the complete (>95%) reduction of Tc^VII^ by NP-magnetite. The *k* ^3^-weighted EXAFS spectra (Figure S4B) show a systematic evolution from the kinetic series to the CoPrec samples (best visible in the region 6–8 Å^–1^). The corresponding Fourier transform magnitudes (Figure S4C) reveal this transition by a Tc–Tc peak at 2.3 Å in the Fourier transform magnitude for pH 5.0, 1 day, and increasingly expressed Tc–Fe peaks at 2.6 Å and 3.0 Å, while the oxygen coordination sphere remains similar for all samples.

To identify the different spectral contributions in the XAS spectra, and hence the different Tc species in the samples, we conducted principal component analysis using the ITFA software package^31^ on all 18 samples as shown in Figure S4. Both XANES and EXAFS spectra of the magnetite samples were reasonably reproduced by two principal components (red lines in Figure S4). Iterative transformation target test, using the ITT module of ITFA, was then used to derive component fractions (Table S5). The deviation of the non-normalized ITFA fractions from the normalized fractions (sum scaled to unity) suggests an uncertainty of the speciation below ∼10%.

Since the EXAFS spectra represent a combination of two spectral components, they are difficult to analyze by shell fitting due to the EXAFS-inherent limited resolution of individual shells (0.13 Å for a chi range of 2–14 Å^–1^, from $\Delta R∼\frac{\pi }{2\Delta k}$ ). Therefore, we employed VARIMAX and ITT modules of the ITFA package to extract the EXAFS spectra of the two endmember components (Figure 2).

**Figure 2:**
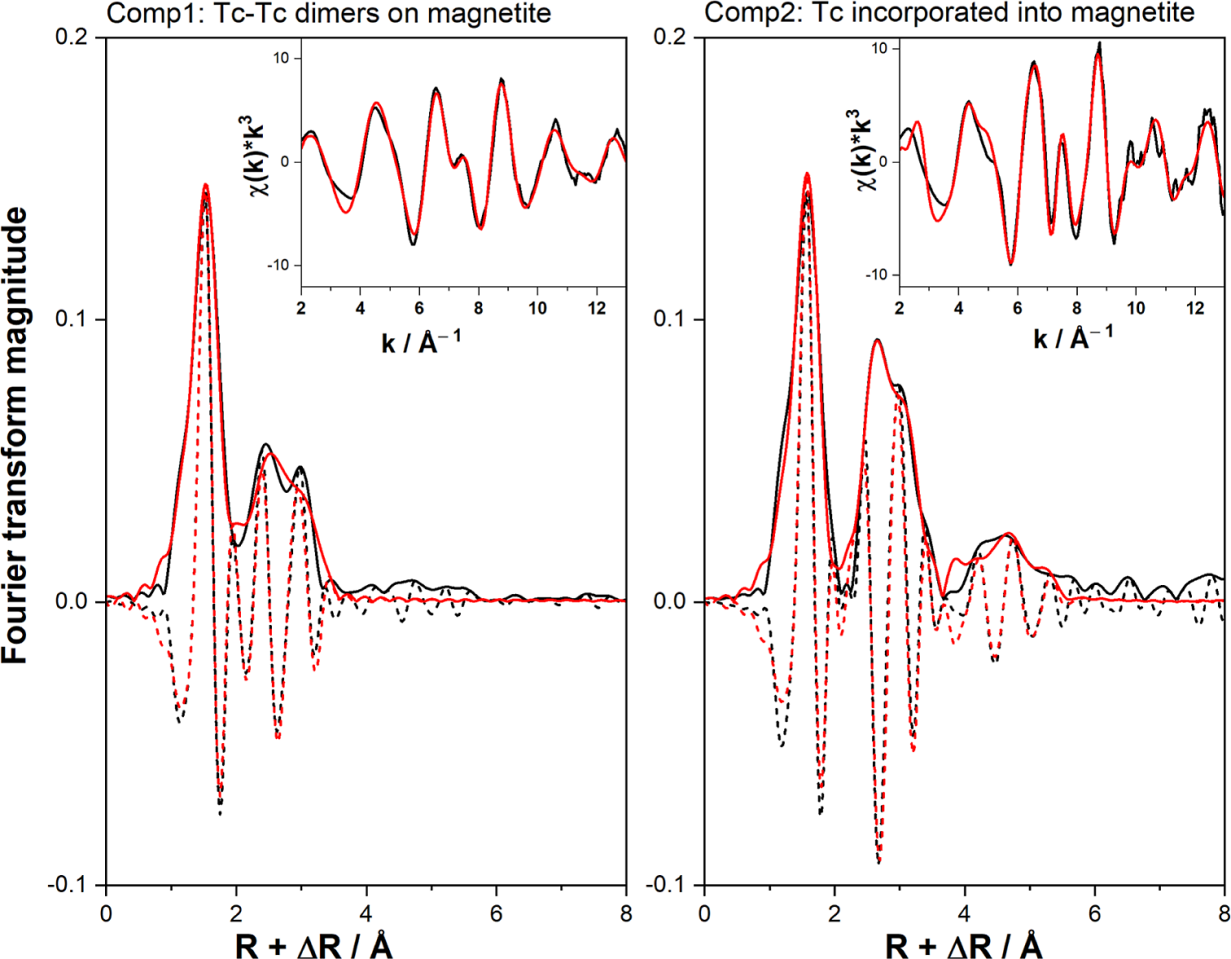
ITFA extracted single-component spectra (black lines) of the endmembers and their reproduction by shell fitting (red lines). Dashed lines represent the imaginary part. Insets show the corresponding *k* ^3^ weighted EXAFS χ­(*k*) spectra. Component 1 (comp 1, left) corresponds to Tc^IV^–Tc^IV^-dimers attached to magnetite, while component 2 (comp 2, right) represents the structurally incorporated Tc^IV^ in magnetite.

The spectrum of the first component (comp 1) corresponds to the spectrum of the Tc^IV^–Tc^IV^-dimers associated with the magnetite surface, similar as reported in.^8^ The spectrum presents a backscattering component at ∼2.5 Å, which can be attributed to the Tc–Tc distance as present in Tc^IV^O_2_·*x*H_2_O that is commonly found in the literature.^36–39^ In the past, this short distance has been attributed to Tc–Fe as well,^30,40^ but it is deemed too short for any kind of known structures in recent literature to our knowledge. Likewise, two backscattering shells at ∼3.1 Å and ∼3.5 Å are observed. These are common distances for the Tc–Fe_oct_ and Tc–Fe_tetr_ shells in magnetite.^8,10,20^ The low intensity of these shells suggested a lower coordination number as demonstrated in the fit (Table 1). Therefore, the shell fit results confirm that the Tc^IV^–Tc^IV^-dimers are attached to the magnetite surface rather than being incorporated. No significant contributions at radial distance >3.5 Å were observed, which indicates a lack of Tc polymerization to longer chains as proposed for the isolated Tc^IV^O_2_·*x*H_2_O^39^ and mixed Tc–Fe chains as suggested by previous EXAFS and DFT studies.^40^

**Table 1:** EXAFS Shell Fit Parameters for Component 1^a^

component 1: Tc^IV^–Tc^IV^-dimers (EXAFS)				TcO_2_·*x*H_2_O fresh (EXAFS)^39^	Tc^IV^–Tc^IV^-dimers (EXAFS)^8^	β-TcO_2_·2H_2_O chains (DFT)^39^
shell	CN	R/Å	σ/Å^2^	R/Å	R/Å	R/Å
Tc–O1	6*	2.00	0.0046	2.01	2.02	1.98
Tc–O2				2.39		2.23
Tc–Tc1	1*	2.53	0.0079	2.55	2.57	2.53
Tc–Fe1	2.5	3.07	0.0079/		3.12	
Tc–Fe2	2.5	3.51	0.0079/		3.52	
Tc–Tc2						4.60

a Coordination numbers fixed to their nominal values are marked with an asterisk (*S*_0_^2^ = 0.9, *E*_0_ = −4.4 eV). For comparison, the EXAFS shell fit distances of a fresh Tc^IV^O_2_·*x*H_2_O and the DFT-derived β-TcO_2_·2H_2_O from Oliveira et al.^39^ fit errors: CN: ±25%; *R*: 0.01 Å, σ^2^: 0.002 Å^2^

Figure 2, right, shows the spectrum of the second component (comp 2). The spectral features and the distances between atoms determined by shell fitting correspond to Tc^IV^ occupying the Fe_oct_ site of magnetite (see Table 2). This is supported by the 6-fold O-coordination and the distance of 2.04 Å (2.06 Å for Fe–O in magnetite). The Tc–Fe1_oct_ distance of 3.11 Å is 0.14 Å (and hence significantly) longer than the Fe–Fe_oct_1 distance in magnetite (2.97 Å). By contrast, the Tc–Fe_tetr_1 distance (3.50 Å) is only slightly longer in comparison to the Fe–Fe_tetr_1 distance in magnetite (3.48 Å). These modifications might be indicative of a local distortion due to the required charge compensation mechanism by replacing Fe^II^ or Fe^III^ by Tc^IV^ (Figure 3), as further investigated by DFT below. In contrast to previous studies, we could fit three additional shells, constituting the longer-range order of magnetite up to 5.6 Å (Table 2). The atomic distances for these shells were all within the expected error range and fit well to the structure of magnetite. A consistent fit was obtained by fixing the coordination numbers of these paths to their crystallographic values. Therefore, the EXAFS fit shows that the Tc^IV^ local environment consists of a full coordination sphere of (magnetite) Fe atoms out to ∼5.6 Å; hence, the average Tc^IV^ is embedded within the magnetite particles to a depth of at least ∼5.6 Å. This interpretation is based on the completeness of the surrounding Fe coordination rather than a direct surface-sensitive measurement.

**Figure 3:**
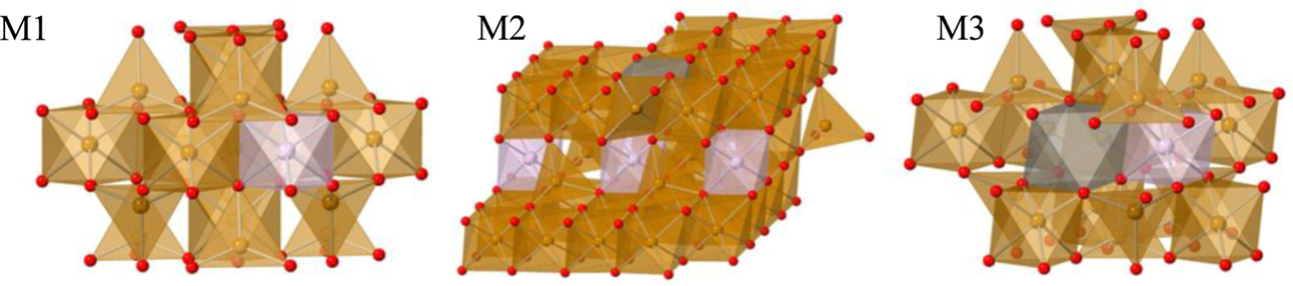
Tc^IV^-substituted magnetite corresponding to mechanisms mentioned above (M1, M2, M3). The structures were refined by DFT. Brown is Fe, pink is Tc, and gray is a vacancy.

### Charge Compensation Mechanisms for Tc^IV^-Substituted Magnetite

While several studies have proven the incorporation of Tc^IV^ into the magnetite structure, none of them discussed the fundamental point of charge compensation via structural changes. Recently, Tc^IV^ incorporation into the magnetite structure via the (111) surface has been theoretically investigated.^41^ The authors investigated charge compensation of the incorporation by distributing the charge among all octahedral Fe. It was found that depending on the oxygen potential, this process can happen via the formation of a vacancy next to the Tc incorporation position or by reducing neighboring octahedral Fe^III^. It is also evident that the exact model of magnetite surface plays a deciding role on whether these energies become negative (favorable) or positive (unfavorable).

We employed DFT calculations to further investigate possible structures of Tc^IV^-substituted magnetite and especially the associated charge compensation mechanism required for replacing Fe_oct_ with a formal oxidation state of +2.5 by Tc^IV^. We tested the following three charge compensation mechanisms, referred to as M1, M2, and M3, with □ being a structural vacancy

M1 $$\left[{Fe}_{3}^{II}{Fe}_{3}^{III}\right]\left({Fe}_{3}^{III}\right)O_{12}+{Tc}^{IV}\to \left[{Tc}^{IV}{Fe}_{4}^{II}{Fe}^{III}\right]\left({Fe}_{3}^{III}\right)O_{12}+{Fe}^{III}$$

M2 $$3\left[{Fe}_{3}^{II}{Fe}_{3}^{III}\right]\left({Fe}_{3}^{III}\right)O_{12}+{3Tc}^{IV}\to 3\left[{Tc}^{IV}{□}_{1/3}{Fe}_{3}^{II}{Fe}_{5/3}^{III}\right]\left({Fe}_{3}^{III}\right)O_{12}+4{Fe}^{III}$$

M3 $$\left[{Fe}_{3}^{II}{Fe}_{3}^{III}\right]\left({Fe}_{3}^{III}\right)O_{12}+{Tc}^{IV}\to \left[{Tc}^{IV}□{Fe}^{II}{Fe}_{3}^{III}\right]\left({Fe}_{3}^{III}\right)O_{12}+2{Fe}^{II}$$

In M1, one Tc^IV^ atom replaces an octahedral Fe^III^ site while another Fe^III^ is reduced to Fe^II^. In M2, three Tc^IV^ atoms replace four Fe^III^ atoms, creating a vacancy. Finally, in M3, one Tc^IV^ atom replaces two Fe^II^ atoms by creating a vacancy. It should be noted that only the Tc^IV^ incorporation is included in these mechanisms, not the reduction process of Tc^VII^O_4_^–^, which was theoretically investigated by Bianchetti et al.^42^

The fully optimized structures are shown in Figure 3, while their interatomic distances are shown in Table 2 along with the distances obtained by experimental EXAFS shell fitting. The interatomic distances calculated with DFT were in good agreement with the values obtained via EXAFS and show the significantly increasing distance of Tc–Fe1_oct_. The distance change of Fe_tetr_1 excludes the M2 structure as determined by the distances derived from DFT. Otherwise, the DFT calculations showed no significant differences between interatomic distances for the M1, M2, and M3 products; therefore, the distances alone were not sufficient to determine which mechanism was the most favored. Additionally, the structures were used to derive simulated EXAFS spectra using the FEFF code (version 9.64). While the differences are minor, M2 shows the highest difference to the isolated Tc^IV^-substituted magnetite component and M3 fits best. A detailed list of interatomic distances can be found in Table S6, a more detailed description of simulating EXAFS spectra and their discussion in Section S6, as well as a comparison in Figure S6.

**Table 2:** EXAFS Shell Fit Parameters for Component 2, Tc Structurally Incorporated in Magnetite^a^

component 2: Tc structurally incorporated in magnetite (EXAFS)				magnetite (XRD)^27^	M1 (DFT)	M2 (DFT)	M3 (DFT)
shell	CN	R/Å	σ/Å^2^	R/Å	R/Å	R/Å	R/Å
Tc–O	6*	2.04	0.0049	2.06	2.05	2.06	2.03
Tc–Fe_oct_1	5*	3.12	0.0090	2.97	3.05	3.06	3.06
Tc–Fe_tetr_1	6*	3.50	0.0110	3.48	3.49	3.55	3.48
Tc–O	24*	4.77	0.0141	4.71	4.73	4.80	4.72
Tc–Fe_oct_2	12*	5.20	0.0131	5.14	5.16	5.24	5.14
Tc–Fe_tetr_2	8*	5.65	0.0142	5.45	5.50	5.57	5.50

a Coordination numbers fixed to their crystallographic values are marked with an asterisk (*S*_0_^2^ = 0.9, *E*_0_ = 5.09 eV). For comparison, the interatomic distances of pure magnetite as derived from pXRD^27^ are shown, as well as the DFT-derived distances for three charge compensation scenarios of TcIV-substituted magnetite (M1, M2, and M3). Fit errors: CN: ±25%; *R*: 0.01 Å, σ^2^: 0.002 Å^2^.

The Tc incorporation energies calculated in function of oxygen chemical potential (Figure S5) suggest M3 as the most favorable mechanism and M2 as the least favorable. The energy curve for M3 is located below zero for the entire range of oxygen potential displayed, becoming more favorable as the oxygen potential increases, going from oxygen-poor (reducing) to oxygen-rich (oxidizing) conditions. On the other hand, the M1 and M2 energy curves are located above zero energy, becoming less favorable as the conditions become more oxidizing. These trends are consistent with the results by Katheras et al.,^41^ although they have not investigated M2. Details on the energy calculations can be found in Section S6.

### Time- and pH-Dependent Tc Speciation

Once the main Tc species in magnetite were identified, we determined their fractions by using VARIMAX rotation and the iterative target test procedure of ITFA. For this, we fixed the fraction of Tc^IV^–Tc^IV^-dimers to unity of Comp 1. All other fractions were freely fitted (Figure 4). The CoPrec sample with the lowest amount of Tc (0.6 kppm) represented the purest endmember of Tc^IV^-substituted magnetite. With increasing Tc loading in CoPrec, Tc^IV^-substituted magnetite remained dominant but Tc^IV^–Tc^IV^-dimer contribution increased, being dominant at 50 kppm loading (5 wt %). This trend was observed in CoPrec samples except for 10 kppm and 20 kppm loadings, which probably resulted from the synthesis being conducted at a later time, suggesting a high experimental variance even though conditions were kept as similar as possible. The amount of incorporated Tc^IV^ into magnetite was calculated from the speciation distribution and the amount of Tc, yielding that the maximum amount of Tc^IV^ to be incorporated is about ∼15 kppm (1.5 wt %) (Table S1). Previous work has demonstrated that Tc^IV^ can be incorporated into in situ-formed, nonstoichiometric magnetite at levels up to 1.86 wt %.^43^ Our measured upper incorporation limit of ∼1.5 wt % (with a maximum calculated value of 1.78 wt %, albeit a large associated error) is consistent with these earlier findings.

**Figure 4:**
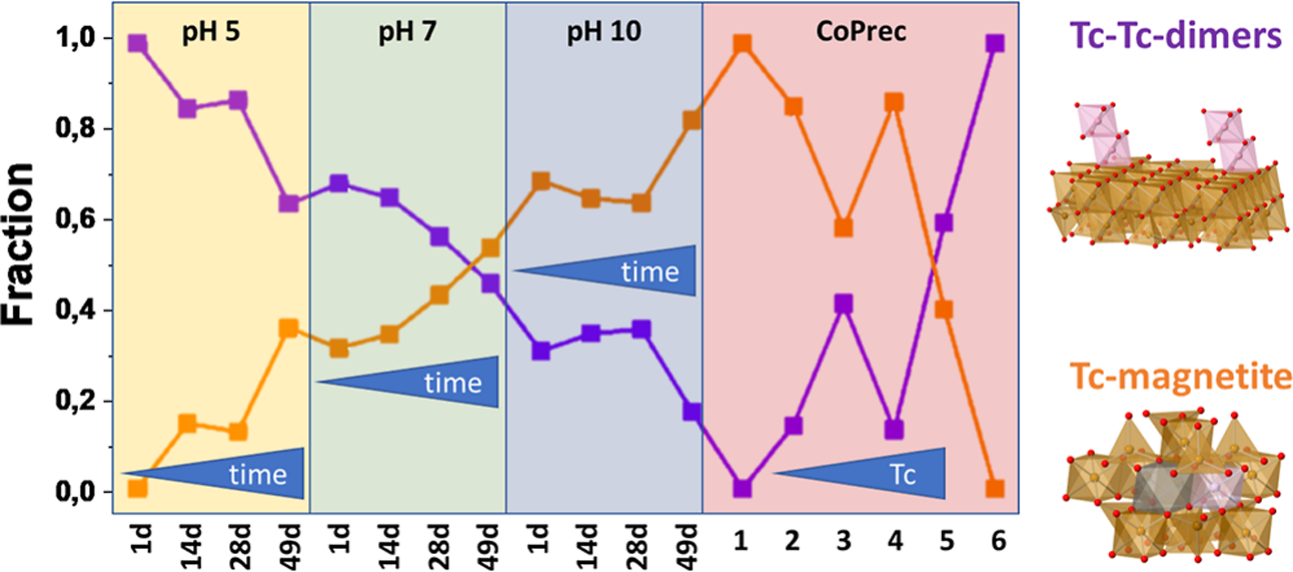
Species distribution of Tc^IV^–Tc^IV^-dimers sorbed on magnetite (purple) and Tc^IV^-substituted magnetite (orange) for Tc sorption on magnetite at different pH (5.0, 7.0 and 10.0) and contact times (7, 14, 28, and 49 days) and in Tc coprecipitates in magnetite (CoPrec, Table S1) with increasing Tc loading; numbers correspond to number in Table S1.

The Tc species distribution of the kinetic series varies with time and pH. At pH 10.0, formation of Tc^IV^-substituted magnetite prevails (83%). After 1 day, more than 60% of Tc^IV^ is already incorporated. In contrast, at pH 5.0, Tc^IV^–Tc^IV^- dimers formed initially but changed with time to 64% Tc-substituted magnetite after 49 days. The behavior at pH 7.0 was intermediate: the amount of the substitution species is at 34% after 1 day and increased only slowly to 53% after 49 days. Overall, in all investigated kinetic series, Tc^IV^-substituted magnetite appears to be the final and, therefore, the thermodynamically most favored product. At pH 5.0, the kinetics are slow enough to show the initial formation of Tc^IV^–Tc^IV^-dimers, which then are slowly converted to Tc^IV^-substituted magnetite. This indeed shows that while Tc is removed from the aqueous phase rapidly, the subsequent structural transformation toward incorporated Tc species proceeds more slowly, as reflected by the time-dependent EXAFS spectra.

### Formation of Tc^IV^-Substituted Magnetite through Coupled Sorption/Redox Reaction

As pointed out, Tc^IV^ incorporation in magnetite occurred at depths >5.6 Å below the magnetite surfaces (Table 2). Lattice diffusion at room temperature is considered to be extremely slow even at temperatures above 1000 °C, even though redox reactions and cation vacancies frequently observed in nanoparticles may accelerate this process.^44^ The question to be answered is hence this: How can Tc^IV^ be incorporated in a thermodynamically stable phase within time scales of a few hours? Several hypotheses are possible.

First, incorporation of Tc^IV^ in magnetite could be favored by higher magnetite solubility and subsequent reprecipitation, which was an hypothesis previously suggested.^8^ According to this hypothesis, the maximum of Tc^IV^ incorporation in magnetite would be at low pH values, when the magnetite solubility is higher. However, in our experiments, Tc^IV^ incorporation is faster at pH 10.0 than at pH 5.0 (Figure 4), which contradicts the hypothesis of Yalcintas et al.^8^

Second, magnetite oxidation to maghemite is considered to proceed through Fe^2+^ diffusion from the crystal lattice to the surface and subsequently to the aqueous phase;^45,46^ this process was observed in magnetite after 24 h at 120 °C.^46^ Likewise, magnetite and Co-doped-ferrite showed similar Fe^2+^ exchange rates,^47^ and since the latter has a 6 orders of magnitude lower electron conductivity, electron diffusion seems to be an unlikely process for the Fe^2+^ exchange between the crystal lattice and the surrounding water, thereby providing circumstantial evidence that ion diffusion may not be the relevant process.

Third, Tc^IV^-incorporation in magnetite could be driven by dissolution/reprecipitation of the solid phase triggered by redox processes in which magnetite would act as a supplier of electrons (redox conveyor belt model of Scherer^47–52^ ). In this model, Fe^2+^ in solution sorbs on magnetite. By subsequently donating one of its electrons to magnetite, the sorbed Fe^2+^ oxidizes to Fe^III^, thereby expanding the structure. Due to the high electron conductivity in the magnetite lattice, the electron is then transferred to another structural Fe^III^ atom on the other side of the particle, which is reduced to Fe^II^ and released as Fe^2+^ into solution. As a consequence, a new layer of Fe-oxide forms at the site where Fe^2+^ originally sorbed while a layer is dissolving on the other side of the particle. This is the most feasible explanation for the observed rapid Tc^IV^ incorporation. Support comes from the obstruction of this process at low pH, in which the formation of a maghemite layer acts as a barrier for the electron and Fe^II^.

### Formation of Tc^IV^–Tc^IV^-Dimers Favored by Maghemitization

Magnetite is known to leach Fe^II^ at low pH, undergoing a phase transition to maghemite ([□Fe_5_^III^]­(Fe_3_^III^)­O_12_; □ is a vacancy) at pH < 7.0, the so-called maghemitization.^45^ The release of structural Fe^II^ creates vacancies in the magnetite structure leading to minor structural reordering and formation of maghemite. We observed at pH 7.0 and 10.0 very little or no Fe^2+^ release (Figure 1), respectively, leaving all structural Fe^II^ available for the fast reduction of Tc^VII^. However, at pH < 6.5, the higher amount (and the onset at higher pH) of Fe^2+^ release in comparison with Fe^3+^ is in line with maghemitization. Maghemitization is further supported by a color change of the suspensions at pH < 3.0 from black magnetite to dark brown maghemite due to the loss of the strong and wide intervalence charge transfer band at about 1500 nm.^53^ Note that the structure of maghemite is very similar to that of magnetite and can be discriminated from magnetite only by a small decrease of the unit cell dimensions by X-ray diffraction (Δ*a* = 0.05 Å; ≈0.6%).^22,54–56^ This diffraction shift was difficult to measure for NPs due to the broadening of diffraction peaks; hence, we were only partially able to verify the process directly by XRD.

At pH 5.0, Fe^II^-leaching happened within minutes to hours on the surface of NP-magnetite (Figure 1), forming a maghemite layer around a core of magnetite.^57^ When a protective outer layer of Fe^III^ and the structural vacancies, i.e., maghemite, are formed, they hinder Fe^II^ diffusing to the surface and leaching in the solution.^46^ Indeed, by using XPS, we could observe a decrease of surface Fe^II^ at pH < 7.0 (Figure S7). This was also the case when using higher amounts of Tc as the Fe^II^ is consumed for Tc^IV^ reduction. The 10 kppm Tc CoPrec sample contained only 13% Fe^II^, while sorption samples at pH > 7.0 had 18–19% Fe^II^ in the near-surface layer probed by XPS. Also, pXRD results showed a minimal shift of the diffraction peak to higher angles at a higher amount of Tc (Table S9), indicating a decrease of the unit cell consistent with maghemitization (Figure S8). Since we assumed that electron transfer from the magnetite structure (band gap between 0 and 1 eV depending on the crystallographic direction^58^) is responsible for the Tc^VII^ reduction, the Fe^III^ passivation layer blocked the electron transfer, thereby kinetically limiting the conveyor belt mechanism that we assumed was responsible for the structural incorporation of Tc^IV^. Instead, the reduced Tc^IV^–Tc^IV^-dimers formed, most likely by a local oversaturation effect. If we assumed that the inhibited electron conduction from the magnetite structure was responsible for the lack of Tc^IV^ structural incorporation, Tc^VII^O_4_^–^ still needs to be reduced to Tc^IV^. The absence of Tc^VII^O_4_^–^ reduction at pH < 5.0 showed that the dissolved Fe^2+^ alone, which is increasingly available with decreasing pH (up to 6 mM), was not able to proceed with such a homogeneous redox reaction, as had been observed before.^40^ The rather small pH range, where Tc^IV^–Tc^IV^-dimers prevail, suggests that it is rather Fe^2+^ initially released from the structure, but then readsorbed at the surface, which leads to the reduction of an oxidant like Tc^VII^O_4_^–^, a process previously observed in Fe^II^-oxides^9,10,59^ and in Fe^II^-sorbed clays^60,61^ for selenite.

### Environmental Relevance

It is commonly assumed that sorption reactions at the mineral water interface lead solely to the electrostatic or chemical attachment of ions to the mineral surface. Even if sorption may lead to the development of mono-, bi-, and sometimes tridentate chemical bonds (depending mostly on ionic charge), this attachment should be readily reversible, in case the chemical/thermodynamic equilibrium changes, leading to a rapid release of the sorbed ion.^62^ Release of ions from a solid mineral phase requires in contrast the dissolution of the mineral phase, or diffusion through the crystal lattice, which depending on its solubility can be many orders of magnitude slower.^44^

In this context, rapid incorporation of an ion by the lattice of a relatively poorly soluble mineral, magnetite, through rapid surface sorption, has fundamental implications for the sequestration of this ion. Our results demonstrate that after interaction of Tc^VII^O_4_^–^ with NP-magnetite, Tc^IV^-substituted magnetite forms fast and is thermodynamically more stable than initially formed Tc^IV^–Tc^IV^-chains sorbed on magnetite.^11,13^ Furthermore, the high incorporation capacity of NP-magnetite (up to 1.5 wt % Tc) highlights its strong scavenging potential. This has important consequences for the safety assessment of nuclear waste management, where an efficient sequestration of Tc must be guaranteed for at least hundred thousand years. Note that magnetite, if not already naturally present in the host rock, will form readily by anoxic corrosion of the steel containers used for waste containment.^12^

In addition to the rapid formation of Tc^IV^-substituted magnetite, our data show that a substantial fraction of Tc^IV^–Tc^IV^-dimers persists even after 49 days. This persistence indicates that Tc immobilization does not proceed exclusively through full structural incorporation but rather through a combination of lattice substitution and dimeric Tc species associated with the magnetite surface or near-surface region. Such dimers may differ in their long-term redox stability and susceptibility to oxidative remobilization compared to fully incorporated Tc^IV^ and therefore need to be considered in long-term safety assessments. While more dedicated reoxidation experiments are ultimately required to quantify remobilization risks, establishing the speciation and distribution of Tc^IV^ between surface-bound and incorporated forms is a crucial first step toward predicting the Tc behavior in changing repository-relevant environments.

Our work suggests the redox conveyor belt model investigated in detail by Scherer and coauthors^47–52^ as the most feasible mechanisms responsible for Tc^IV^ incorporation in magnetite. The required charge compensation occurs via substitution of two Fe^II^ atoms by a Tc^IV^ and formation of a vacancy. While diffusion into nanopores has been revealed as possible mechanism for a relatively fast uptake into the interior of mineral particles,^63^ we demonstrate here for the first time that coupled ion/electron exchange (conveyor belt model) can lead to the structural incorporation without requiring (nano)­pores or structural defects common in nanoparticles.^64^ While this process has been previously identified by employing two different Fe isotope ratios and Mössbauer spectroscopy for a rapid exchange of Fe lattice ions in various Fe oxides, we believe that this is the first time that a similar process is identified for the incorporation of an element (Tc), which is normally not present in the mineral host.

The surprisingly fast Tc^IV^ incorporation in magnetite has implications for a rapid incorporation of other pollutants in a presumably stable mineral phase, potentially preventing pollutant migration in general. If the only requirements for this incorporation are a redox reaction between an Fe^II^-bearing mineral and a reducible species whose reduced state ionic radius in octahedral coordination is similar to the cations of the host phase, then pollutant incorporation should be observable for other cations with similar properties, like Cr, Cu, or V. Peterson et al.,^65^ for instance, studied the sorption of Cr^VI^ to magnetite and found reduction to Cr^III^ with Cr-metal distances (not able to distinguish between Fe and Cr neighbors) at about 3 Å and CN of 3.2–4.4. They interpreted these EXAFS fit data with the formation of a tridentate inner-sphere surface complex or formation of small surface clusters but excluded incorporation by magnetite because of relatively low CN. In comparison to other studies, however, the observed CN is actually quite high and, together with relatively high Debye–Waller factors, might also be interpreted as a bulk incorporation process similar to our observation. While plotted EXAFS spectra in this first paper do not provide evidence for longer Cr–Fe distances, which would further support bulk incorporation, a follow-up paper^66^ seems to indicate such distances, but without providing fits. It might be worthwhile to revisit the redox-driven uptake of metals by magnetite in general with this mechanism in mind.

## Supplementary Material



## Notes

Characterization of magnetite nanoparticles; sample lists of sorption and coprecipitation experiments; STEM–EDX analysis; XAS data treatment and experimental parameters; ITFA analysis; DFT calculations; XAS simulation; XPS; and pXRD (PDF)

## +

Applied Geochemistry, Institute of Earth and Environmental Sciences, University of Freiburg, 79104 Freiburg im Breisgau, Germany

## #

Institute of Physics, Carl von Ossietzky Universität Oldenburg, Carl-von-Ossietzky-Str. 9–11, 26129 Oldenburg, Germany.
