# Supplementary material for: XAFS and DFT Insights into the Kinetics and Mechanisms of Technetium Reduction by Nanoparticulate Magnetite
Source: Environ Sci Technol. 2026 Jun 23;60(26):18844–53. doi: 10.1021/acs.est.6c01654 (PMC13348022; doi:10.1021/acs.est.6c01654)
Supplement: Supplementary file 1 [file es6c01654_si_001.pdf]

Supporting Information: XAFS and DFT insights into the kinetics and mechanisms of technetium reduction by nanoparticulate magnetite

Thomas Zimmermann[1,2,3], Natalia Mayordomo[1], Augusto F. Oliveira[1,4], Felix Brandt[5], Martina Klinkenberg[5], Juri Barthel[5], Dieter Schild[6], Kerstin Hockmann[3], Thorsten Stumpf[1], Andreas C. Scheinost[2].

[1]Helmholtz-Zentrum Dresden-Rossendorf, Institute of Resource Ecology, Bautzner Landstraße 400, 01328 Dresden, Germany

[2]The Rossendorf Beamline at ESRF – The European Synchrotron, CS40220, 38043 Grenoble Cedex 9, France

[3]Current affiliation: Applied Geochemistry, Institute of Earth and Environmental Sciences, University of Freiburg, 79104 Freiburg im Breisgau, Germany

[4]Current affiliation: Institute of Physics, Carl von Ossietzky Universität Oldenburg, Carl-von-Ossietzky-Str. 9–11, 26129 Oldenburg, Germany

[5]Institute of Fusion Energy and Nuclear Waste Management (IFN), Forschungszentrum Jülich GmbH, 52428 Jülich, Germany

[6]Institute for Nuclear Waste Disposal, Karlsruhe Institute of Technology, P.O. Box 3640, D-76021 Karlsruhe, Germany

Catalogue of Supporting Information

S1 Characterization of magnetite nanoparticles....4

S2 Sample lists and experimental parameters for Tc-magnetite....6

S3 Aqueous analytics....10

S4 STEM-EDX FZJ....12

S5 X-ray absorption spectroscopy (XAS)....14

S6 DFT Calculations....18

S7 SEM and XPS....26

S8 Powder X-ray diffraction (pXRD) of CoPrec....28

S9 References....30

Table of Content

Table S1: Experimental details of the coprecipitation experiments. 7

Table S2: Experimental details of the batch sorption experiments of the pH series (Tc-pH). Equilibration time was 4 weeks, [magnetite] = 4 g/l, $[Tc^{VII}]_{ini}$ = 24.27 µM for all samples. 8

Table S3: Experimental details of the batch sorption experiments of the kinetic series (Tc-kin). 8

Table S4: Experimental details of the batch sorption experiments of the short kinetic series (Tc-s-kin). 9

Table S5: Species distribution of ITFA. In the last two columns total concentrations of fixed to 1, while in the others it is not, therefore the Sum is adding the concentrations together. The difference to 1 indicates the error of the algorithm. 17

Table S6: Distances from the DFT structures for the three different mechanisms. In mechanism 2, there are 3 different Tc atoms. For Mechanism 1 it can be seen, that for each shell the distances are the same for each atom, while in M2 and M3, the distances vary within each shell due to distortion leading to a higher debye-waller factor. 19

Table S7: Calculation of amount of magnetite/maghemite by pXRD for 3 different reflexes....28

Figure S1: pXRD of pure NP-magnetite (black) with Reference PDF-04-012-7038 (red) from database PDF4 from ICDD 5
Figure S2: TEM images of pure NP-magnetite at different magnifications........6
Figure S3: STEM EDX results for sample CoPrec-6. (A) High-angle annular dark-field (HAADF) image of the magnetite nanoparticles (bright) on a thin carbon support film (dark). (B) Fe-K map and (C) Tc-L map of net intensities after background subtraction. (D) X-ray emission spectra, integrated from an area containing particles (black) and from an area containing no particles (grey) over the energy range covering the Tc-L and the Fe-K emission series. The spectra were scaled relative to each other compensating for differences in the integration areas. 13
Figure S4: Tc K-edge spectra. A) XANES, B) EXAFS and C) its respective Fourier transform. Numbers in A and B correspond to the numbers in Table S5. In C the experimental parameters are given again for clarity. 15
Figure S5: Formation energies for the Tc incorporation into magnetite in function of the variation in the oxygen chemical potential $\Delta\mu_O$ for the different incorporation mechanisms. $\Delta\mu_O$ values are limited to $\Delta\mu_O \leq 0$, with the upper bound corresponding to the most oxygen-rich conditions (oxidizing) and lower values corresponding to oxygen-poor conditions (reducing). 22
Figure S6: Simulated XAS from M1, M3 and averaged M2 (for the three different Tc). Upper normalized absorptions spectra, bottom k^3 weighted chi. 24
Figure S7: SEM image of Sample CoPrec-3 ([Tc]=10 kppm) used for XPS. The morphology of all samples looked similar. 26
Figure S8: XPS spectra for A) Fe2p, B) Tc 3d and C) O 1s of 5 samples: CoPrec3 (red), Tc-pH-3 (blue), Tc-pH-6 (green), Tc-pH-9 (orange) and Tc-pH-13 (cyan). Sample details can be found in Table S1 and Table S2. 27
Figure S9: Diffraction data of pure magnetite (black) and CoPrec (2-red, 3-blue, 4-green, 5-pink, 6-brown) for different sections of relevant peaks indicating transformation of magnetite to maghemite.29

S1 Characterization of magnetite nanoparticles

Quantification by powder X-ray diffraction (pXRD)

Solids were analyzed by pXRD (MiniFlex 600 powder XRD, by Rigaku) using Cu Kα (λ=1.54184 Å) as X-ray source, that has an X-ray generation of 40 kV / 15 mA (600 W). The spectrum was recorded in scan continuous mode. The sample preparation was carried out inside a glovebox, where a wet paste was mounted on an airtight sample holder covered with Kapton foil (Rigaku) to ensure the inert conditions during the measurement. The data analysis software used was PDXL: integrated x-ray powder diffraction software, Version 2.8.1.1, Rigaku.

The magnetite synthesis yielded pure particles (Figure S1) with a size of approximately 15 nm in diameter. Synthesis could be scaled down without any change in the resulting product for smaller batches, where parameters can be changed to see their effect on the particle size of magnetite. The particle size was confirmed for a few samples via transmission electron microscopy (TEM), Figure S2. The particle size was estimated using the Scherrer equation (Eq.1) via pXRD. The results for all samples were within error range.

$$D = \frac{k * \lambda}{FWHM * \cos \Theta} \qquad \textbf{Eq.1}$$

D … Mean particle size in nm for particle diameter < 200 nm

k … Formfactor (for round shape approx. 0.9)

λ … X-ray wavelength (Cu Kα = 0.154059 nm)

FWHM … Full width at half maximum (corrected with Si single crystal measurement)

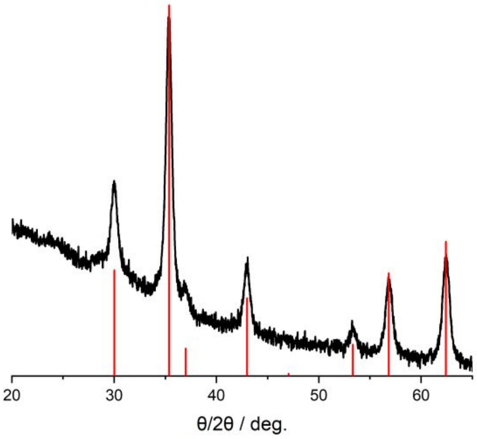

Figure S1: pXRD of pure NP-magnetite (black) with Reference PDF-04-012-7038 (red) from database PDF4 from ICDD

Θ … Angle of reflex (35.43° $\Theta/2\Theta$) in radian measure

Analysis by transmission electron microscopy (TEM)

TEM measurements were carried out using a FEI Tecnai G2 microscope operated at 200 kV, located at the Fusion Laboratory of the Institute for Applied Materials at KIT. The instrument is equipped with an in-situ Gatan Tridiem camera for high-resolution imaging and an energy dispersive X-ray spectroscopy (EDX) detector. Electron diffraction patterns obtained from the samples were analyzed and compared using the Process Diffraction software[1]. For sample preparation, a few microliters of solids suspended in ethanol were deposited onto a TEM grid and dried under an argon atmosphere.

TEM results indicate a single phase, coherent with magnetite structure with diameters on average 12.8 ± 2.8 nm. The particles form large agglomerates but are not compact.

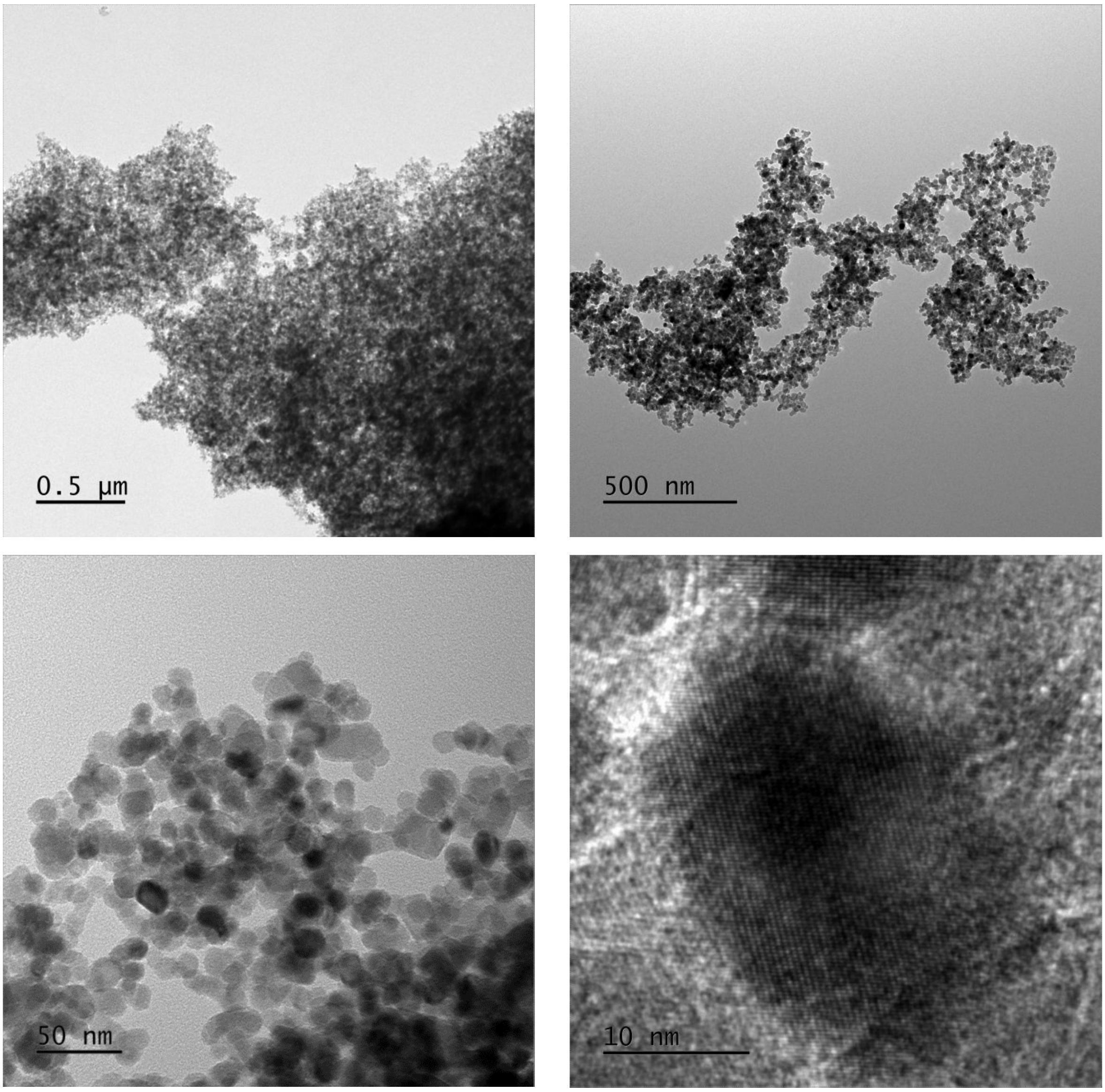

Figure S2: TEM images of pure NP-magnetite at different magnifications.

S2 Sample lists and experimental parameters for Tc-magnetite

For all samples in tables S1-S3 in this paragraph, the following numbers near the sample name indicate the measurement by corresponding analytic method. Not all results are shown.

1… X-ray absorption spectroscopy

2… X-ray photoelectron spectroscopy

3… powder X-ray diffraction

4… Electron microscopy

Table S1: Experimental details of the coprecipitation experiments.

Sample CoPrec	[Tc] (ppm)	$[Tc]_{aq,ini}$ (µM)	Tc-Magn $(\%)_{EXAFS}$	$Magh_{theo}$ (%)	$[Tc]_{inc}$ (kppm)	pH_{eq}
1[1,3]	600	24.3	100	0.4	0.6	8.7
2[1,3]	3000	121	90	2.1	2.7	9.2
3[1,2,3]	10000	404	64	7	6.4	9.7
4[1,3,4]	20000	809	89	14	17.8	9.5
5[1,3,4]	30000	1213	55	21	16.5	9.8
6[1,3,4]	50000	2022	22	35	11.0	10.1

$Magh_{theo}$ is a theoretical calculation of oxidation of magnetite to maghemite occurring due to the reduction of Tc^{VII} to Tc^{IV}. Reduction of Tc needs 3 e^-, Fe^{II} to Fe^{III} yields 1 e^- with Fe_3O_4 = 0%, γ-Fe_2O_3 = 100%.

pH_{eq} is the pH that is when the solid was kept with fresh supernatant after washing (pure magnetite yielded 8.4)

Tc-Magn$(\%)_{EXAFS}$ was obtained from the species distribution in EXAFS analysis for Tc-substituted magnetite (see REF

$[Tc]_{inc}$ is calculated from the EXAFS species distribution as the actual amount incorporated by magnetite

Table S2: Experimental details of the batch sorption experiments of the pH series (Tc-pH). Equilibration time was 4 weeks, [magnetite] = 4 g/l, $[Tc^{VII}]_{ini}$ = 24.27 µM for all samples.

Sample Tc-pH	pH_{ini}	pH_{end}	Eh (mV)	$[Tc]_{aq}$ (µM)	sorbed Tc (%)	$[Fe^{2+}]_{aq}$ (mM)	$[Fe_{tot}]_{aq}$ (mM)
1[3]	2	1.9	674	24.82[b]	0*	6.16	13.42
2[3]	3	2.9	499	24.42[b]	0*	4.71	6.94
3[1,2,3]	4	5.1	104	0	100	2.69	3.15
4[1,3]	5	5.2	105	0	100	2.17	2.42
5[1,3]	6	5.6	44	0	100	0.92	0.91
6[1,2,3]	7	6.5	-64	0	100	0.16	0.16
7[1,3]	8	7.5	-183	0	100	0	0
8[1,3]	9	9.0	-265	0	100	0	0
9[1,2,3]	10	9.7	-264	0	100	0	0
10[1,2]	11	10.9	-331	0.02	99.9	0	0
11[1,2]	12	11.9	-365	0.02	99.9	0	0
12[3]	13[a]	12.6	-413	0.17	99.3	0	0
13[1,2,3]	14[a]	12.7	-433	0.41	98.3	0	0

[a]… pH$_{ini}$ only by adjustment of NaOH in matrix, not measured

[b]… slightly more Tc present in solution than 100 % but within error of LSC

pH_{end} pH were adjusted after 1d, 3d, 7d and then every week, given value is after 4 weeks

$[Fe^{2+}]_{aq}$: Ferrozine method (see section S3) for Fe^{2+}, $[Fe_{tot}]_{aq}$ by ICP

Table S3: Experimental details of the batch sorption experiments of the kinetic series (Tc-kin).

Sample Tc-kin	pH_{ini}	pH_{end}	Eh (mV)	time (days)	sorbed Tc (%)	$[Fe^{2+}]_{aq}$ (mM)	$[Fe_{tot}]_{aq}$ (mM)
1[1]	5	5.55	112	1	100	0.35	0.35
2[1]	5	5.38	98	14	100	2.15	2.15
3[1]	5	5.20	103	28	100	1.85	2.26
4[1]	5	5.28	95	49	100	3.63	4.98
5[1]	7	6.85	-128	1	100	0.04	0.04
6[1]	7	6.94	-83	14	100	0*	0.01
7[1]	7	6.60	-71	28	100	0.12	0.14
8[1]	7	6.66	-141	49	100	0.40	0.44
9[1]	10	9.82	-348	1	100	0	0
10[1]	10	9.95	-273	14	100	0	0
11[1]	10	10.00	-225	28	100	0	0
12[1]	10	10.03	-391	49	100	0	0

$[Fe^{2+}]_{aq}$: Ferrozine method (see section S3) for Fe^{2+}, $[Fe_{tot}]_{aq}$ by ICP

Table S4: Experimental details of the batch sorption experiments of the short kinetic series (Tc-s-kin).

Sample Tc-s-kin	pH_{ini}	pH	time (min)	sorbed Tc (%)
1	5	5.3*	15	85.7
		4.99	30	97.1
		5.10	60	99.9
		5.26	120	100
		5.80	240	100
2	7	7.3*	15	94.1
		6.93	30	98.2
		6.98	60	99.1
		7.07	120	100
		6.95	240	100
3	10	10.2*	15	99.0
		10.01	30	100
		9.98	60	100
		9.97	120	100
		9.98	240	100

The pH-electrode after 15 min was not yet equilibrated (indicated by *) as the pH was still dropping on the pH-meter. This is due to experimental constraints when preparing the samples before measuring pH. Afterwards the drift at pH 5 is real with barely any changes at pH 7 and 10.

S3 Aqueous analytics

Measurements of pH and redox potential (Eh)

The pH was adjusted by adding either NaOH or HCl in concentrations of 1 M, 0.1 M and 0.01 M. The pH measurements were carried out after calibration (pH buffers 4.006, 6.865 and 9.180) using a pH meter (pH3110, WTW) and a pH electrode (SI Analytics Blue Line). The pH was measured in suspensions without agitation until drifts were less than 0.02 /min, but with a maximum of 15 min waiting time. Eh was measured after ensuring a proper functionality of the electrode (Pt with Ag/AgCl; InLab Redox Micro, Mettler Toledo) with a redox buffer solution (220 mV / pH 7). Eh measurements were also performed in suspensions without agitation. Due to drifting values, Eh values were taken after 15 min of equilibration. The measured Eh values were converted to hydrogen standard electrode by adding +207 mV.

Quantification of Tc by liquid scintillation counting (LSC)

0.25 ml of supernatant were mixed with 5 ml of scintillation cocktail (Ultima Gold™, Perkin Elmer) and placed in the liquid scintillation counter (1414 LSC Winspectral α/β Wallac, Perkin Elmer; detection limit: 2.5 cpm; measuring time: 10 min) to determine Tc in solution. Since basically all Tc is removed from the supernatant, it is impossible to calculate a K_D value according to Eq.2, since $[Tc]_t$ would be 0. The detection limit of 2.5 cpm would be equal to a concentration of 2.68 nmol/l. Assuming the initial concentration of 24.3 µmol/l of Tc in 50 ml containing 200 mg magnetite, the log K_D can be said to be higher than 6.35 ml/g.

$$K_D = \frac{[\mathrm{Tc}]_0 - [\mathrm{Tc}]_t}{[\mathrm{Tc}]_t} * \frac{V}{m} \qquad \textbf{Eq.2}$$

$[Tc]_0$... Initial technetium concentration in Bq/mL

$[Tc]_t$... Technetium concentration after a certain time in Bq/mL

V... Sample volume in ml

m... Mass of solid in g

Quantification of elements by inductively coupled plasma mass spectrometry (ICP-MS)

Element concentrations of the supernatants after ultracentrifugation were measured using inductively coupled plasma mass spectrometry (ICP-MS) with a NexION 350x from Perkin Elmer. The samples were acidified using concentrated HNO_3.

Quantification of dissolved Fe by "Ferrozine method"[2]

Fe^{2+} and Fe^{3+} contents of the supernatant were quantified directly or indirectly, respectively, by complexing Fe^{2+} with ferrozine and consecutively using UV/VIS spectroscopy for determination of the ratio. Fe^{2+} forms a purple complex with ferrozine, which was indicated by a color change of the solution. To avoid saturation of the detector, highly concentrated solutions were diluted (solutions were basically black in such a case). The concentration of the Fe^{2+}-ferrozine complex has its maximum absorbance at 562 nm. An UV/VIS spectrometer (AvaSpec-ULS2048 StarLine, Avantes) was located outside the glovebox along with the lamp (AvaLight-DH-S-BAL, Avantes) and both of them were connected to the cell holder inside the glovebox using optical fiber.

For the analysis, a ferrozine stock solution was prepared (1 g/l ferrozine in 50 mM HEPES buffer, pH 7). Two times 125 µl supernatant was taken after centrifugation of the sample at 6000 rpm for 30 min. The Fe^{2+} content was analyzed by directly adding 1 ml of ferrozine stock to these 125 µl (Sample A). The Fe^{3+} content was analyzed by taking 125 µl of supernatant with 0.5 ml of a 0.5 g/l $Na_2S_2O_3$ solution added to reduce Fe^{3+} to Fe^{2+} and then analyzed in the same way (Sample B). Sample A gives $[Fe^{2+}]$, Sample B $[Fe(total)]_{aq}$ and thus $[Fe^{3+}]$ can be calculated according to Eq.3.

$$[Fe^{3+}] = [Fe(total)]_{Sample\ B} - [Fe^{2+}]_{Sample\ A} \qquad \textbf{Eq.3}$$

When measuring UV/VIS, the absorption band of the ferrozine complex is a rather broad signal which causes a higher error in determining the concentrations. ICP-MS is a much more sensitive tool to quantify Fe. To avoid this error, the total concentration of Fe was measured by ICP-MS. UV/VIS helped to measure the amount of Fe^{2+} and see the increase by reducing Fe^{3+}. From this the ratio of Fe^{2+}/Fe^{3+} was taken and multiplied by the total $[Fe]_{aq}$ measured by ICP-MS.

S4 STEM-EDX FZJ

Scanning transmission electron microscopy (STEM) and energy-dispersive X-ray spectroscopy (EDX) elemental mapping were performed using a TFS Spectra 300 microscope (Thermo Fisher Scientific, Netherlands), operated at an accelerating voltage of 200 kV and a beam current of 230 pA. The focused STEM probe was generated with a convergence semi-angle of 27.5 mrad and corrected for spherical aberration. High-angle annular dark-field (HAADF) images were recorded using a detector covering scattering angles between 62 and 200 mrad, enabling Z-contrast imaging. In this imaging mode, higher intensity corresponds to regions with higher atomic number, greater specimen thickness, or increased material density.[3]

EDX elemental maps were acquired during scanning with a Super-X EDX detector (Thermo Fisher Scientific, Netherlands)[4] offering a maximum collection angle of 0.7 sr, to qualitatively determine the elemental distribution within the SAL and the surrounding material. The spectrometer operated with a dispersion of 5 eV per channel, covering X-ray energies up to 20 keV, and data acquisition was synchronized with the scan using a dwell time of 10 µs. Total acquisition times for the mappings ranged between 20 and 30 minutes, and empirical background correction was applied. Approximately one quarter of the detector area was shadowed by the sample holder, resulting in an effective solid angle for EDX detection of about 0.5 sr.

In general, the particles did not change in appearance to those of pure magnetite (section S1). They indicate a single phase, coherent with magnetite structure with average diameters of approximately 15 nm. The particles form large agglomerates but are not compact. The STEM EDX mappings show a low Tc-signal in general, only in the areas covered by nanoparticles. Due to the low Tc signal no clear conclusion can be drawn from the mappings about whether Tc is uniformly or non-uniformly distributed in the magnetite. The spectra shown in Fig. S3D indicate that there is no Tc signal in areas not containing particles. The spurious intensity at such areas occurring in the map shown in Fig. S3C can be attributed to noise. That the Tc-L signal mapping essentially correlates with the Fe-K map, supports conclusions drawn from the XAS data.

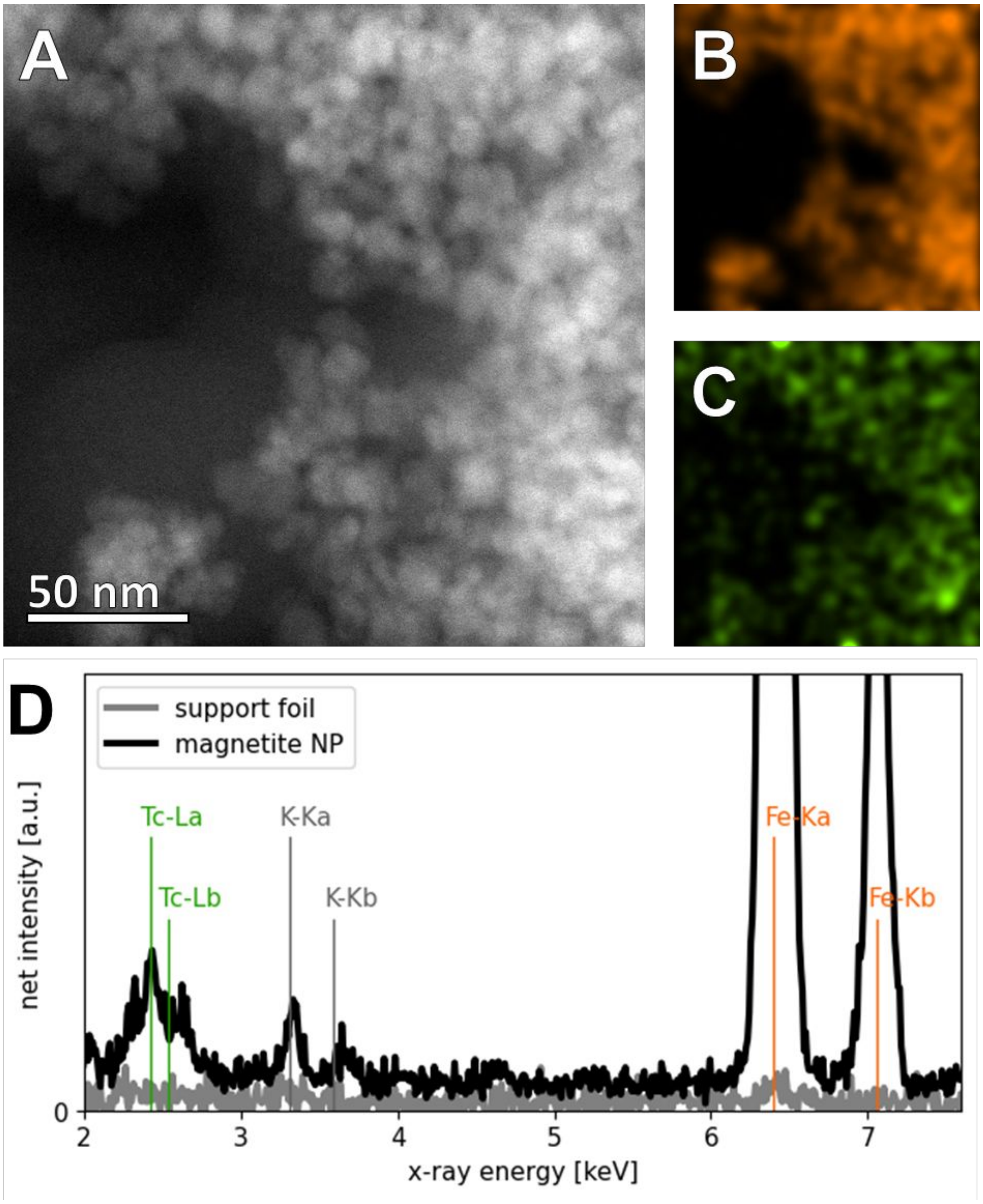

Figure S3: STEM EDX results for sample CoPrec-6. (A) High-angle annular dark-field (HAADF) image of the magnetite nanoparticles (bright) on a thin carbon support film (dark). (B) Fe-K map and (C) Tc-L map of net intensities after background subtraction. (D) X-ray emission spectra, integrated from an area containing particles (black) and from an area containing no particles (grey) over the energy range covering the Tc-L and the Fe-K emission series. The spectra were scaled relative to each other compensating for differences in the integration areas.

S5 X-ray absorption spectroscopy (XAS)

The Tc-containing wet pastes were mounted on double (for Tc load of 600 ppm) or quadruple sealed (for Tc loads > 600 ppm) high-density polyethylene sample holders inside the glovebox for XAS measurements. The samples were immediately flash-frozen with liquid nitrogen when taken out of the glovebox and stored in a liquid nitrogen container for storage and transportation to prevent further aging and oxidation of the samples.

Samples were measured at the Rossendorf Beamline (BM20) at the European Synchrotron Radiation Facility (ESRF) in Grenoble, France[5]. Tc K-edge (21 044 eV) XAS spectra were acquired in fluorescence mode. The energy of the Si(111) double crystal monochromator was calibrated using a Mo foil (edge energy 20 000 eV). Two Rh-coated mirrors were used to collimate the beam onto the first monochromator crystal. Fluorescence spectra were collected with an 18-element, high-purity, electrically-cooled solid-state Ge detector (Ultra-LEGe, GUL0055, Mirion Technologies) with digital spectrometer (XIA Falcon-X). Further details are reported elsewhere[5]. During the measurement, the samples were kept at 15 K within a closed cycle He cryostat to avoid photon-induced changes of oxidation state and to reduce thermal disorder.

Normalization, transformation from energy into k-space, subtraction of a spline background, and shell fits were performed with WinXAS following standard procedures.[6,7] All fits were carried out in R-space (1−5.5 Å) of k^3-weighted spectra (2−13 $Å^{-1}$) using theoretical backscattering amplitudes and phase shifts calculated with FEFF 9.64 on clusters (R_{max} = 8 Å) derived from magnetite[8] and TcO_2[9] structures. For the structural models, Tc was placed into the central 6-coordinated Fe position to simulate Tc-doped magnetite. The Debye−Waller factor was restricted to float between 0.001 and 0.02 $Å^2$. Furthermore, spectra were analyzed by the iterative transformation factor analysis (ITFA) software package developed by Rossberg et al.[10] Shortly, the derivation of the number of spectral components is based on three factors, the minimum of the Malinowski indicator value calculated for all principal components, a visual inspection of the principal components to discriminate the ones that contain the extended X-ray absorption fine-structure (EXAFS) signal from those that arise from fluctuations of the spline background removal and noise, and finally, and perhaps most important, the reconstruction of the experimental data by a minimum number of components. Varimax rotation and iterative transformation target test modules are then used to identify the spectral endmembers and to extract their EXAFS spectra. A detailed description of these modules can be found elsewhere[10]. The XANES white line position was identical for all samples and aligned with Tc^{IV} (Figure S4). Since the pre-edge peak of Tc^{VII} would be

easily recognizable above the flat pre-edge background of Tc^{IV}, we can estimate that at least 98 % of solid-associated Tc is reduced from (VII) to (IV).

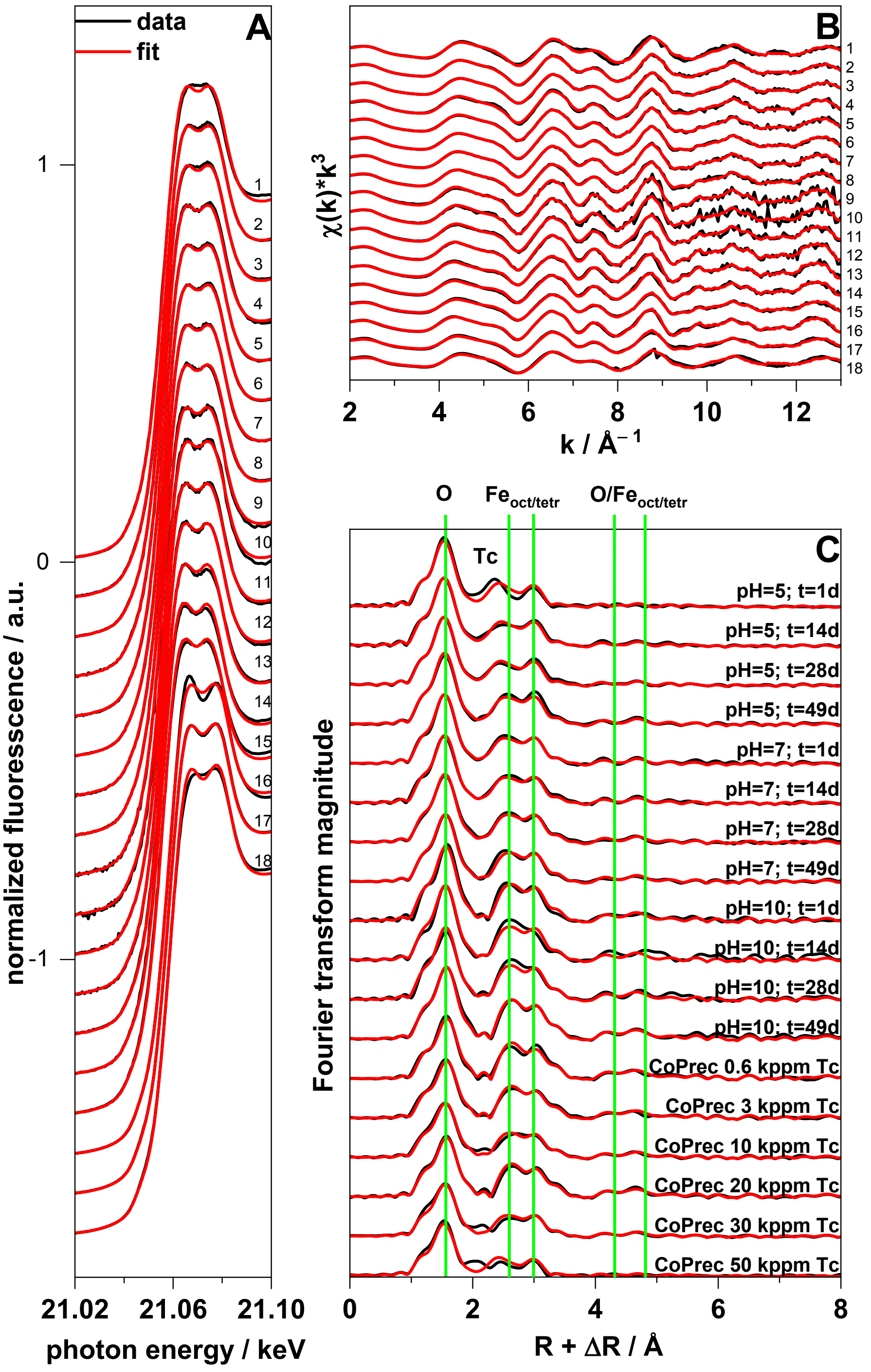

Figure S4: Tc K-edge spectra. A) XANES, B) EXAFS and C) its respective Fourier transform. Numbers in A and B correspond to the numbers in Table S5. In C the experimental parameters are given again for clarity.

Table S5: Species distribution of ITFA. In the last two columns the fractions are scaled to provide a sum of unity, while in the others it is not. The deviation of the unscaled sum from unity provides an error estimate.

No. ITFA	Sample	Tc^{IV}-Tc^{IV}-dimers	Tc-substitued magnetite	Sum	Tc^{IV}-Tc^{IV}-dimers	Tc-substitued magnetite
1	Tc-kin-1	1.00	0.00	1.00	1.00	0.00
2	Tc-kin-2	0.91	0.17	1.08	0.85	0.15
3	Tc-kin-3	0.94	0.13	1.07	0.88	0.12
4	Tc-kin-4	0.76	0.40	1.16	0.66	0.34
5	Tc-kin-5	0.78	0.33	1.11	0.70	0.30
6	Tc-kin-6	0.72	0.38	1.10	0.66	0.34
7	Tc-kin-7	0.63	0.50	1.13	0.56	0.44
8	Tc-kin-8	0.53	0.58	1.11	0.48	0.52
9	Tc-kin-9	0.37	0.86	1.23	0.30	0.70
10	Tc-kin-10	0.54	0.71	1.25	0.43	0.57
11	Tc-kin-11	0.44	0.77	1.21	0.36	0.64
12	Tc-kin-12	0.19	1.00	1.19	0.16	0.84
13	Tc-CoPrec-1	0.00	1.00	1.00	0.00	1.00
14	Tc-CoPrec-2	0.10	0.84	0.94	0.11	0.89
15	Tc-CoPrec-3	0.37	0.53	0.90	0.41	0.59
16	Tc-CoPrec-4	0.12	0.86	0.98	0.12	0.88
17	Tc-CoPrec-5	0.48	0.38	0.86	0.56	0.44
18	Tc-CoPrec-6	0.80	0.00	0.80	1.00	0.00

S6 DFT Calculations

DFT calculations were used to assess three different Tc incorporation mechanisms. In mechanism 1, the Fe^{II} charge balanced substitution, an octahedral Fe^{III} site of magnetite is replaced with a Tc^{IV} atom, leading to the reduction of a second Fe^{III} site to Fe^{II}, which can be represented as

$$(Fe^{III}_2)[Fe^{II}_2Fe^{III}_2]O_8 + Tc^{IV} \longrightarrow (Fe^{III}_2)[Fe^{II}_3Tc^{IV}]O_8 + Fe^{III} \qquad \textbf{Eq.4}$$

In mechanism 2, the Fe^{III} vacancy-driven substitution, three Tc^{IV} replace four octahedral Fe^{III} sites, thereby creating a vacancy

$$(Fe^{III}_4)[Fe^{II}_4Fe^{III}_4]O_{16} + 3\ Tc^{IV} \longrightarrow (Fe^{III}_4)[Fe^{II}_4Tc^{IV}_3\square]O_{16} + 4\ Fe^{III} \qquad \textbf{Eq.5}$$

In mechanism 3, the Fe^{II}-vacancy substitution, a single Tc^{IV} replaces two octahedral Fe^{II} sites, also producing a vacancy

$$(Fe^{III}_2)[Fe^{II}_2Fe^{III}_2]O_8 + Tc^{IV} \longrightarrow (Fe^{III}_2)[Fe^{III}_2Tc^{IV}\square]O_8 + 2\ Fe^{II} \qquad \textbf{Eq.6}$$

All DFT calculations were conducted with the AMS/BAND 2022 program[11,12] using the PBE density functional[13] and numerical atomic orbitals (NAOs) augmented with triple-zeta polarized (TZP) sets of Slater-type basis functions. Scalar relativistic effects were included with the zeroth-order regular approximation (ZORA) model[14]. All structures were fully optimized, including the lattice parameters of the crystals. The initial spin states of the tetrahedral Fe^{III} sites in magnetite and the corresponding Tc-incorporated structures were flipped to allow the development of a ferrimagnetic arrangement during the calculations.

Magnetite – Fe_3O_4 (Fd-3m). The initial structure of magnetite was obtained from the Materials Project database[15], entry mp-19306. The atomic coordinates were optimized using the default convergence settings of AMS/BAND with the primitive representation of the unit cell, which contains two Fe_3O_4 units. For calculating the final energies, the optimized cell was transformed to its conventional representation (8 Fe_3O_4 units) and the numerical quality was increased from "Normal" to "Good", resulting in a finer sampling of the Brillouin zone.

Tc-substituted magnetite. The initial structures were constructed by replacing octahedral Fe atoms in the magnetite conventional unit cell (8 Fe_3O_4 units) obtained from the Materials Project database[15], entry mp-19306. The lowest possible number of Tc atoms were incorporated to the structure based on the mechanisms represented in Eqs. 4-6 – i.e., one Tc for mechanisms 1 and 3, and three Tc atoms for mechanism 2. Since mechanism 1 does not lead to vacancy formation, there is only one possible structure (note that the reduction of an octahedral Fe^{III} to Fe^{II} will result during the DFT calculation as the electron density of the inserted Tc atom is redistributed within the system). For

mechanisms 2 and 3, all possible relative positions of Tc atoms and vacancies within the unit cells were considered. The Python library Pymatgen[16] was used to exclude equivalent structures, resulting in 14 structures for mechanism 2 and three structures for mechanism 3. All structures were then pre-optimized within the Γ-point approximation while keeping the lattice vectors fixed. Subsequently, the lowest-energy structure obtained for each mechanism was fully optimized (including lattice vectors) using the default numerical quality and convergence criteria of AMS/BAND. At last, the numerical quality was set to "Good" for the calculation of the final energies.

Final structure for mechanism 2. In order to obtain a structure for mechanism 2 with Tc concentration comparable to mechanisms 1 and 3, a 2×2×2 replica of the magnetite primitive cell (16 Fe_3O_4 units) has been used to generate the initial Tc-substituted structure. In this case, we only considered one substitution structure, equivalent to the lowest-energy structure obtained for the smaller magnetite cell (8 Fe_3O_4 units). The new structure was optimized as described above and used for the analyses presented in the paper.

Table S6: Distances from the DFT structures for the three different mechanisms. In mechanism 2, there are 3 different Tc atoms. For Mechanism 1 it can be seen, that for each shell the distances are the same for each atom, while in M2 and M3, the distances vary within each shell due to distortion leading to a higher debye-waller factor.

Mechanism 1		Mechanism 2-Tc1		Mechanism 2-Tc2		Mechanism 2-Tc3		Mechanism 3	
Tc-O	2.049	Tc-O	2.059	Tc-O	2.050	Tc-O	1.979	Tc-O	1.973
Tc-O	2.049	Tc-O	2.061	Tc-O	2.062	Tc-O	2.003	Tc-O	1.973
Tc-O	2.049	Tc-O	2.064	Tc-O	2.068	Tc-O	2.066	Tc-O	2.037
Tc-O	2.049	Tc-O	2.071	Tc-O	2.069	Tc-O	2.078	Tc-O	2.039
Tc-O	2.049	Tc-O	2.071	Tc-O	2.069	Tc-O	2.099	Tc-O	2.075
Tc-O	2.049	Tc-O	2.081	Tc-O	2.070	Tc-O	2.102	Tc-O	2.075
Tc-Fe1	3.050	Tc-Fe1	3.008	Tc-Fe1	3.013	Tc-Fe1	2.929	Tc-□	2.835
Tc-Fe1	3.050	Tc-Fe1	3.032	Tc-Fe1	3.042	Tc-□	2.963	Tc-Fe1	3.020
Tc-Fe1	3.050	Tc-Fe1	3.063	Tc-Fe1	3.082	Tc-Fe1	2.992	Tc-Fe1	3.020
Tc-Fe1	3.050	Tc-Fe1	3.093	Tc-Fe1	3.095	Tc-Fe1	3.008	Tc-Fe1	3.084
Tc-Fe1	3.050	Tc-Fe1	3.102	Tc-Fe1	3.118	Tc-Fe1	3.104	Tc-Fe1	3.084
Tc-Fe1	3.050	Tc-Fe1	3.114	Tc-Fe1	3.133	Tc-Fe1	3.129	Tc-Fe1	3.097
Tc-Fe2	3.486	Tc-Fe2	3.544	Tc-Fe2	3.522	Tc-Fe2	3.502	Tc-Fe2	3.448
Tc-Fe2	3.486	Tc-Fe2	3.547	Tc-Fe2	3.537	Tc-Fe2	3.510	Tc-Fe2	3.448
Tc-Fe2	3.486	Tc-Fe2	3.551	Tc-Fe2	3.548	Tc-Fe2	3.541	Tc-Fe2	3.477
Tc-Fe2	3.486	Tc-Fe2	3.556	Tc-Fe2	3.553	Tc-Fe2	3.551	Tc-Fe2	3.492
Tc-Fe2	3.486	Tc-Fe2	3.560	Tc-Fe2	3.561	Tc-Fe2	3.557	Tc-Fe2	3.492
Tc-Fe2	3.486	Tc-Fe2	3.568	Tc-Fe2	3.566	Tc-Fe2	3.575	Tc-Fe2	3.504

Tc-O	4.715	Tc-O	4.721	Tc-O	4.695	Tc-O	4.730	Tc-O	4.626
Tc-O	4.715	Tc-O	4.742	Tc-O	4.717	Tc-O	4.751	Tc-O	4.626
Tc-O	4.715	Tc-O	4.756	Tc-O	4.744	Tc-O	4.752	Tc-O	4.659
Tc-O	4.715	Tc-O	4.775	Tc-O	4.745	Tc-O	4.767	Tc-O	4.659
Tc-O	4.715	Tc-O	4.787	Tc-O	4.747	Tc-O	4.774	Tc-O	4.677
Tc-O	4.715	Tc-O	4.788	Tc-O	4.763	Tc-O	4.779	Tc-O	4.677
Tc-O	4.715	Tc-O	4.789	Tc-O	4.766	Tc-O	4.782	Tc-O	4.689
Tc-O	4.715	Tc-O	4.793	Tc-O	4.770	Tc-O	4.783	Tc-O	4.689
Tc-O	4.715	Tc-O	4.796	Tc-O	4.771	Tc-O	4.784	Tc-O	4.710
Tc-O	4.715	Tc-O	4.799	Tc-O	4.785	Tc-O	4.797	Tc-O	4.710
Tc-O	4.715	Tc-O	4.802	Tc-O	4.790	Tc-O	4.799	Tc-O	4.723
Tc-O	4.715	Tc-O	4.804	Tc-O	4.794	Tc-O	4.803	Tc-O	4.723
Tc-O	4.735	Tc-O	4.804	Tc-O	4.797	Tc-O	4.806	Tc-O	4.748
Tc-O	4.735	Tc-O	4.805	Tc-O	4.803	Tc-O	4.818	Tc-O	4.748
Tc-O	4.735	Tc-O	4.811	Tc-O	4.803	Tc-O	4.823	Tc-O	4.753
Tc-O	4.735	Tc-O	4.812	Tc-O	4.807	Tc-O	4.823	Tc-O	4.753
Tc-O	4.735	Tc-O	4.820	Tc-O	4.816	Tc-O	4.833	Tc-O	4.762
Tc-O	4.735	Tc-O	4.821	Tc-O	4.816	Tc-O	4.837	Tc-O	4.762
Tc-O	4.735	Tc-O	4.824	Tc-O	4.821	Tc-O	4.839	Tc-O	4.762
Tc-O	4.735	Tc-O	4.825	Tc-O	4.823	Tc-O	4.841	Tc-O	4.762
Tc-O	4.735	Tc-O	4.829	Tc-O	4.827	Tc-O	4.854	Tc-O	4.771
Tc-O	4.735	Tc-O	4.843	Tc-O	4.842	Tc-O	4.868	Tc-O	4.771
Tc-O	4.735	Tc-O	4.853	Tc-O	4.866	Tc-O	4.880	Tc-O	4.783
Tc-O	4.735	Tc-O	4.868	Tc-O	4.866	Tc-O	4.888	Tc-O	4.783
Tc-Fe	5.162	Tc-Fe	5.183	Tc-Fe	5.177	Tc-Fe	5.046	Tc-Fe	4.940
Tc-Fe	5.162	Tc-Fe	5.207	Tc-Fe	5.222	Tc-Fe	5.053	Tc-Fe	4.940
Tc-Fe	5.162	Tc-Fe	5.230	Tc-Fe	5.226	Tc-Fe	5.198	Tc-Fe	5.142
Tc-Fe	5.162	Tc-Fe	5.235	Tc-Fe	5.237	Tc-Fe	5.222	Tc-Fe	5.142
Tc-Fe	5.162	**Tc-□**	5.238	Tc-Fe	5.248	Tc-Fe	5.223	Tc-Fe	5.169
Tc-Fe	5.162	Tc-Fe	5.246	Tc-Fe	5.248	Tc-Fe	5.227	Tc-Fe	5.169
Tc-Fe	5.162	Tc-Fe	5.253	Tc-Fe	5.248	Tc-Fe	5.246	Tc-Fe	5.175
Tc-Fe	5.162	Tc-Fe	5.260	Tc-Fe	5.261	Tc-Fe	5.250	Tc-Fe	5.175
Tc-Fe	5.162	Tc-Fe	5.260	Tc-Fe	5.263	Tc-Fe	5.257	Tc-Fe	5.209
Tc-Fe	5.162	Tc-Fe	5.263	Tc-Fe	5.270	Tc-Fe	5.263	Tc-Fe	5.209
Tc-Fe	5.162	Tc-Fe	5.268	Tc-Fe	5.274	Tc-Fe	5.264	Tc-Fe	5.217
Tc-Fe	5.162	Tc-Fe	5.324	Tc-Fe	5.310	Tc-Fe	5.266	Tc-Fe	5.217
Tc-Fe	5.482	Tc-Fe	5.472	Tc-Fe	5.509	Tc-Fe	5.494	Tc-Fe	5.436
Tc-Fe	5.482	Tc-Fe	5.495	Tc-Fe	5.529	Tc-Fe	5.512	Tc-Fe	5.439
Tc-Fe	5.504	Tc-Fe	5.571	Tc-Fe	5.559	Tc-Fe	5.542	Tc-Fe	5.476
Tc-Fe	5.504	Tc-Fe	5.577	Tc-Fe	5.579	Tc-Fe	5.560	Tc-Fe	5.476
Tc-Fe	5.504	Tc-Fe	5.589	Tc-Fe	5.590	Tc-Fe	5.561	Tc-Fe	5.499
Tc-Fe	5.504	Tc-Fe	5.589	Tc-Fe	5.590	Tc-Fe	5.604	Tc-Fe	5.525
Tc-Fe	5.504	Tc-Fe	5.598	Tc-Fe	5.603	Tc-Fe	5.607	Tc-Fe	5.565
Tc-Fe	5.504	Tc-Fe	5.615	Tc-Fe	5.603	Tc-Fe	5.613	Tc-Fe	5.565

Formation energies of the Tc-substituted species. In order to compare the relative stability of the Tc-incorporated structures, their formation energies ($\Delta_f E$) were computed as a function of the oxygen chemical potential using the expression [17]

$$\Delta_f E = E_{\text{Tc-magnetite}} + (x + n)\mu_{\text{Fe}} - (E_{\text{magnetite}} + x\mu_{\text{Tc}}) \qquad \textbf{Eq.7}$$

where $E_{\text{magnetite}}$ and $E_{\text{Tc-magnetite}}$ are the DFT energies calculated for pure magnetite and Tc-substitute species, x is the number of Tc atoms incorporated into the magnetite lattice and n is the number of vacancies resulting from the incorporation process. The chemical potentials for Fe, Tc, and O are defined as

$$\mu_{\text{Fe}} = \frac{1}{3}\left(\mu_{\text{Fe}_3\text{O}_4} - 4\mu_{\text{O}}\right) \qquad \textbf{Eq.8}$$

$$\mu_{\text{Tc}} = \mu_{\text{TcO}_2} - 2\mu_{\text{O}} \qquad \textbf{Eq.9}$$

$$\mu_{\text{O}} = \frac{1}{2}\mu_{\text{O}_2} + \Delta\mu_{\text{O}} \qquad \textbf{Eq.10}$$

assuming that the system is at thermodynamic equilibrium with a reservoir of bulk Fe_3O_4, a reservoir of bulk TcO_2, and a reservoir of O_2 gas. In this framework, the $\Delta\mu_O$ deviation from the O_2 gas reference relates to the thermodynamic availability of oxygen in the environment and is bound to $\Delta\mu_O \leq 0$, with the upper bound ($\Delta\mu_O = 0$) corresponding to oxygen-rich (oxidizing) conditions, and $\Delta\mu_O < 0$ corresponding to oxygen-poor (reducing) conditions. The $\mu_{Fe_3O_4}$, μ_{TcO_2}, and μ_{O_2} chemical potentials were set to the corresponding DFT energies. In the case of TcO_2, the DFT energy was calculated for the β-TcO_2 phase[9].

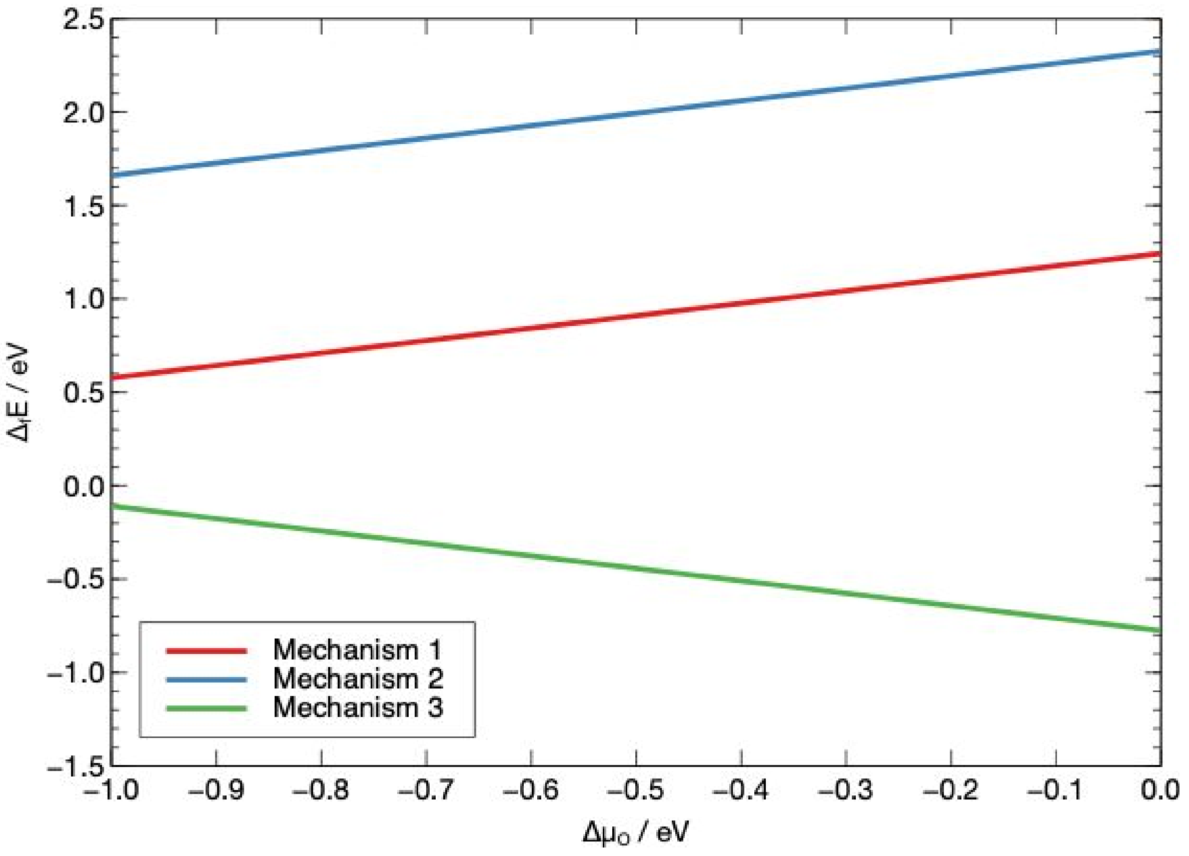

Figure S5: Formation energies for the Tc incorporation into magnetite in function of the variation in the oxygen chemical potential $\Delta\mu_O$ for the different incorporation mechanisms. $\Delta\mu_O$ values are limited to $\Delta\mu_O \leq 0$, with the upper bound corresponding to the most oxygen-rich conditions (oxidizing) and lower values corresponding to oxygen-poor conditions (reducing).

Simulation and comparison of XAS. From the DFT-derived structural models, theoretical EXAFS spectra were generated using the FEFF code (version 9.64) in JFEFF. FEFF was employed to calculate the photoelectron scattering amplitudes, phase shifts, and multiple-scattering paths associated with each absorbing Tc site, applying a DWF of 0.005. For compound M2, three crystallographically distinct Tc environments were present. Therefore, separate FEFF calculations were performed for each unique Tc atom, and the resulting spectra were averaged to obtain the final simulated EXAFS signal. A comparison of the simulated spectra for all three compounds is shown in Figure S6. Additionally, the derived spectra were fitted in the same way as the isolated components using WinXAS (described in Section S5). The resulting fitting parameters can be found in Table S7

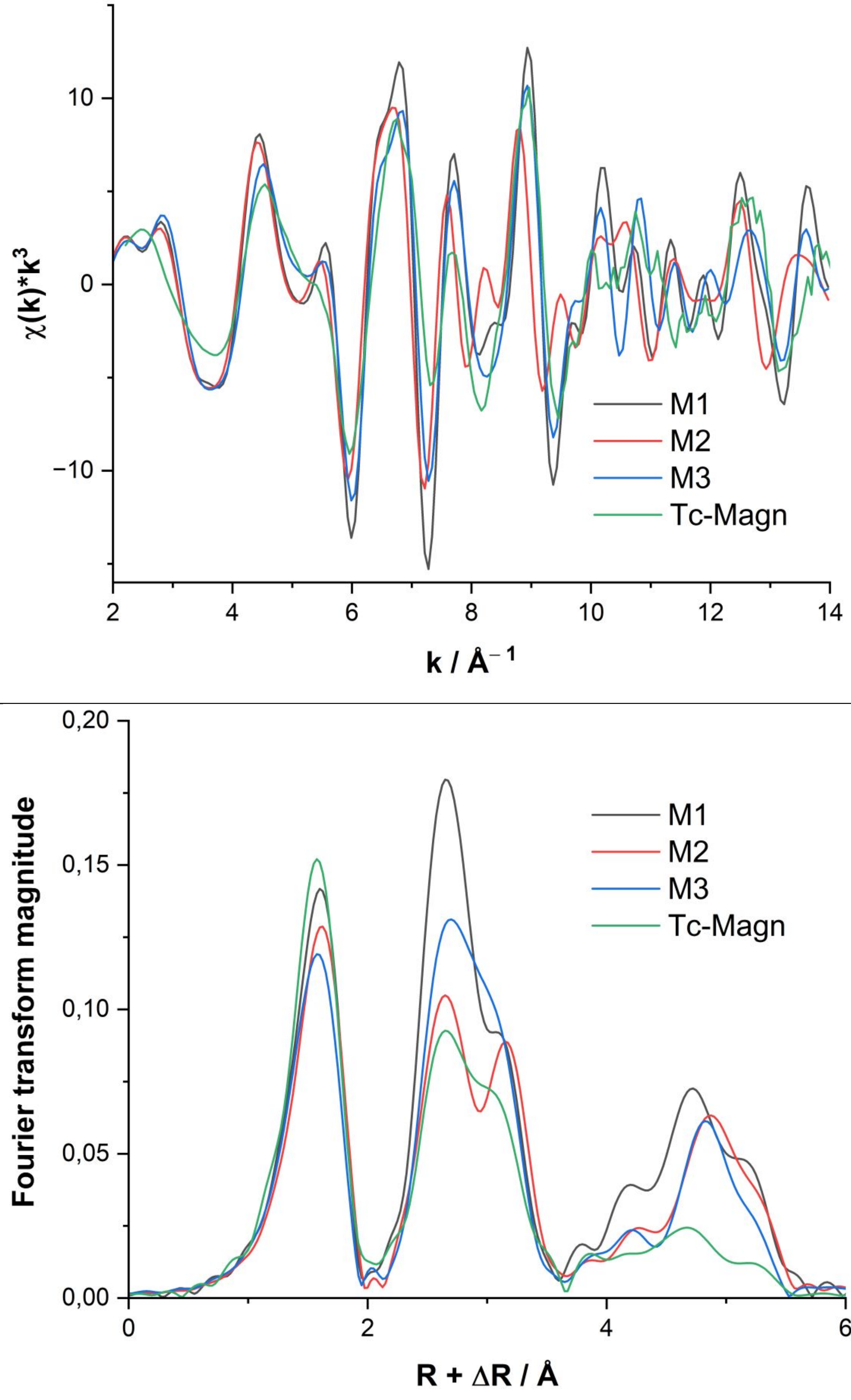

Figure S6: Simulated EXAFS spectra of DFT-refined models M1, M3 and M2 (the latter by averaging the three different Tc sites). Top shows the k^3-weighted EXAFS spectra and bottom their respective Fourier Transforms.

Table S7: Fitting parameters of derived EXAFS spectra for the three mechanisms

**Component 2: Tc structurally incorporated in magnetite (EXAFS)**				**M1 (Sim-Fit)**		**M2 (Fit)**		**M3 (Fit)**	
Shell	CN	R / Å	σ / Å²	R / Å	σ / Å²	R / Å	σ / Å²	R / Å	σ / Å²
Tc-O	6*	2.04	0.0049	2.04	0.0047	2.06	0.0055	2.03	0.0068
Tc-Fe_{oct}1	5*	3.12	0.0090	3.04	0.0039	3.07	0.0074	3.06	0.0061
Tc-Fe_{tetr}1	6*	3.50	0.0110	3.48	0.0063	3.55	0.0066	3.48	0.0069
Tc-O	24*	4.77	0.0141	4.71	0.0066	4.78	0.0113	4.67	0.0131
Tc-Fe_{oct}2	12*	5.20	0.0131	5.14	0.0053	5.23	0.0061	5.18	0.0065
Tc-Fe_{tetr}2	8*	5.65	0.0142	5.54	0.0033	5.60	0.0051	5.53	0.0073

In Figure S6, the Tc^{IV}-substituted magnetite (green) shows a general good agreement with the simulated spectra of all three structural mechanisms. The spectra show a perfect phase alignment until k = 8 $Å^{-1}$. After this, differences are visible with M3 showing the best phase alignment for all maxima and minima. M2 shows big differences, while M3 has a quite good phase alignment but bigger discrepancy in the amplitude. This is even better highlighted in the FT. The general shell distances agree well. For the first Fe_{oct} and Fe_{tetr} shell, the signal is splitting strongly for M2 due to the higher distance of $Fe\text{-}_{tetr,}$ which is not observed for our isolated component. The intensity of the shells aligns best with M2 and worst with M1, which is arising from the effect of DWF due to distortion. M1 does not show this distortion and therefore gives the highest intensities. The differences between the three distinct Tc environments simulates an even higher distortion than for M3. It is important to keep in mind, that we simulate infinitely large single crystalline structures from DFT. In reality we have nanometer sized particles that show surface defects and distortions. It is therefore expected to have higher intensities for the simulations of perfect crystals in comparison to the experimental spectra of real nanoparticles.

To accurately compare fitting of our spectra with the simulated ones, we used the same shells to fit again the structure. Ideally we would get a DWF of 0.005 and the same distances. Due to the error of the fit, small differences are possible but also as we cannot fit all distorted atoms separately, a higher DWF is expected. Here we observe the best match for M3 (closest to isolated component), while the distances all barely change compared to the theoretical structures.

S7 SEM and XPS

An FEI Quanta 650 FEG environmental scanning electron microscope was used to image the sample surfaces. SEM–energy-dispersive X-ray (SEM-EDX) spectra from selected regions were obtained using a Thermo Scientific UltraDry silicon drift X-ray detector (Peltier-cooled) in combination with the NORAN System 7 microanalysis system and Pathfinder analysis software (software version 2.11). Both sample preparation and SEM-EDX measurements were conducted under anoxic conditions. The SEM-images looked similar for all samples (Figure S7).

XPS measurements were conducted using a PHI5000 VersaProbe II system (ULVAC-PHI Inc.) equipped with a monochromatic Al Kα X-ray source (1486.7 eV). Survey spectra of the conductive samples were acquired using an X-ray source power of 32 W and an analyzer pass energy of 187.85 eV. High-resolution spectra of selected elemental peaks were collected at a pass energy of 23.5 eV, corresponding to an energy resolution of 0.67 eV (FWHM) at the Ag $3d_{5/2}$ peak of pure silver. The binding energy scale was calibrated using established reference values for pure metal standards measured with a monochromatic Al Kα source[18] (Cu $2p_{3/2}$ at 932.62 eV, Au $4f_{7/2}$ at 83.96 eV). The uncertainty in the reported binding energies is estimated to be ±0.2 eV.

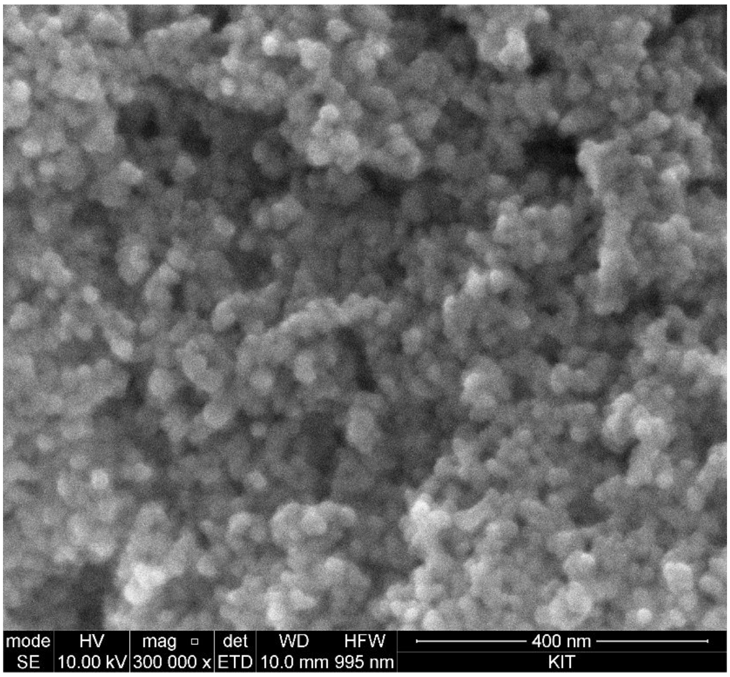

Figure S7: SEM image of Sample CoPrec-3 ([Tc]=10 kppm) used for XPS. The morphology of all samples looked similar.

From XPS measurements (Figure S8), the ratio of Fe(II)/Fe(III) can be calculated[19]. For the ideal magnetite structure this ratio is 33 %. It should be noted that the information depth (95 % of signal) of Fe 2p is only ~2.7 nm and therefore gives a result of the surface and not the complete phase. The CoPrec-3 and Tc-pH-3 sample had a low Fe(II)/Fe(III) ratio of 13 % and 11 % respectively, while Tc-pH-6, Tc-pH-9 and Tc-pH-13 had a Fe(II)/Fe(III) ratio of 18-19 %. This indicates a higher rate of maghemitization at low pH and at higher [Tc]. The binding energies of Tc $3d_{3/2}$ and Tc $3d_{5/2}$ could be detected in all samples with approximately 260.5 eV and 256.5 eV respectively, indicating Tc(IV). Binding energies of O 1s indicate oxidic O. Therefore, the formation of hydroxides can be excluded which would be visible in a shift to around 531.3 eV.

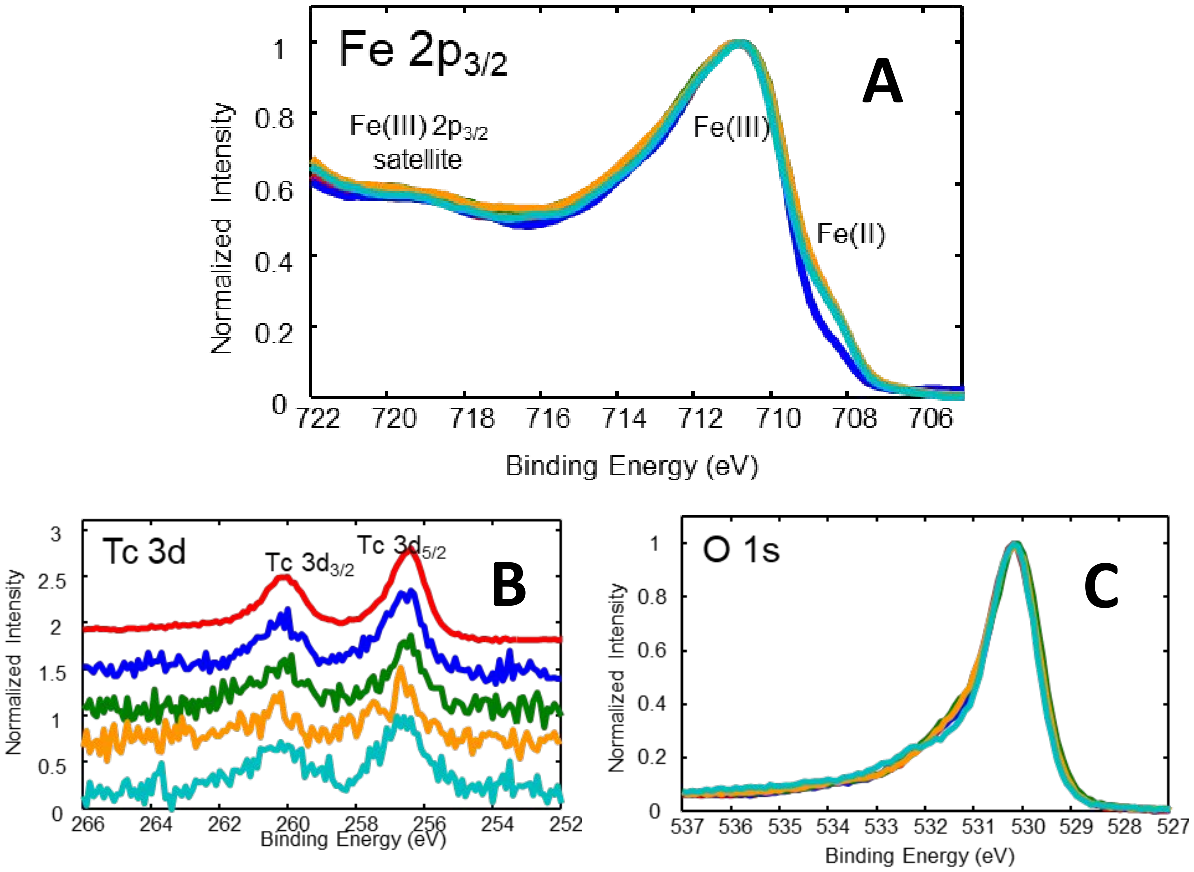

Figure S8: XPS spectra for A) Fe2p, B) Tc 3d and C) O 1s of 5 samples: CoPrec3 (red), Tc-pH-3 (blue), Tc-pH-6 (green), Tc-pH-9 (orange) and Tc-pH-13 (cyan). Sample details can be found in Table S1 and Table S2.

S8 Powder X-ray diffraction (pXRD) of CoPrec

With reflexes of pure magnetite and maghemite it is possible to calculate the mixture assuming a solid-solution using Eq.7. The exact maximum of the diffractions is hard to estimate due to the broadening happening due to the presence of nanoparticles as well as the noise of measurement. The results for three prominent reflexes at approximately 35 °, 57 ° and 62 ° can be found in Table S8. The sections of the diffraction pattern can be found in Figure S9. The sample of Tc-CoPrec3 has indeed higher amount of maghemitization, which controls the amount of Tc-Tc dimers forming on the surface. While it is not clear how this happened experimentally, it follows the inner logic of species distribution via EXAFS analysis. For CoPrec6, there also other reflexes starting to short, indicating other Fe^{III} minerals that formed due to the high amount of oxidation occurring in the Tc reduction process. These phase changes do not influence EXAFS results indicating a phase change occurring from maghemite.

$$\%Magn = \frac{\Theta - \Theta(Magh)}{\Theta(Magn) - \Theta(Magh)} * 100\% \qquad \textbf{Eq.11}$$

Table S8: Calculation of amount of magnetite/maghemite by pXRD for 3 different reflexes

Sample	Reflex ~ 35° / %Magn		Reflex ~ 57° / %Magn		Reflex ~ 62° / %Magn		% Magn
Magnetite	35.328°	100	56.837°	100	62.452°	100	100
CoPrec2	35.416°	71	56.918°	81	62.533°	83	78
CoPrec3	35.519°	37	57.000°	62	62.613°	66	55
CoPrec4	35.429°	67	56.933°	78	62.515°	87	77
CoPrec5	35.450°	60	56.933°	78	62.533°	83	73
CoPrec6	35.566°	21	57.177°	22	62.792°	28	24
Maghemite*	35.630°	0	57.271°	0	62.925°	0	0

*Approximate from literature[20]

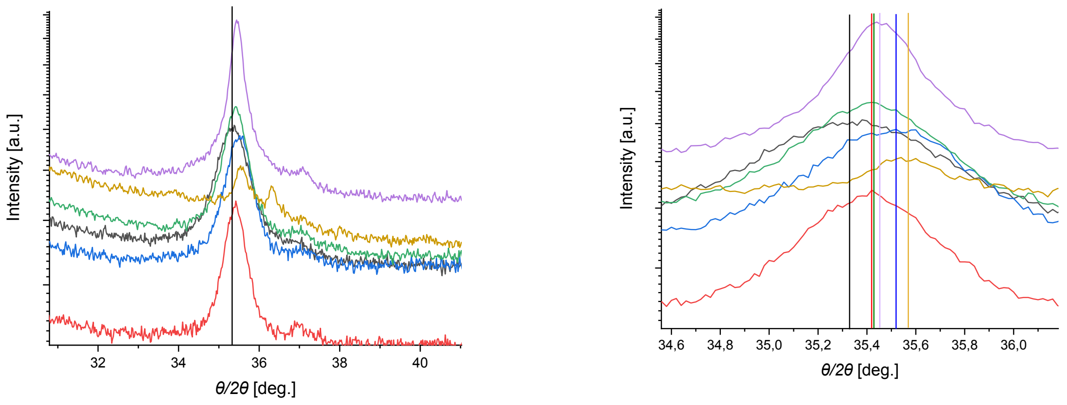

Figure S9: Diffraction data of pure magnetite (black) and CoPrec (2-red, 3-blue, 4-green, 5-pink, 6-brown) for different sections of relevant peaks indicating transformation of magnetite to maghemite.

S9 References

(1) Lábár, J. L. Electron Diffraction Based Analysis of Phase Fractions and Texture in Nanocrystalline Thin Films, Part I: Principles. *Microsc. Microanal.* **2008**, *14*, 287–295. https://doi.org/10.1017/S1431927608080380.

(2) Stookey, L. L. Ferrozine-A New Spectrophotometric Reagent for Iron. *Anal. Chem.* **1970**, *42* (7), 779–781. https://doi.org/10.1021/ac60289a016.

(3) Pennycook, S J; Mayr, G. Z-Contrast Stem for Materials Science. *Ultramicroscopy* **1989**, *143* (1), 82–97.

(4) Schlossmacher, P.; Klenov, D. O.; Freitag, B.; von Harrach, H. S. Enhanced Detection Sensitivity with a New Windowless XEDS System for AEM Based on Silicon Drift Detector Technology. *Micros. Today* **2010**, *18* (4), 14–20. https://doi.org/10.1017/s1551929510000404.

(5) Scheinost, A. C.; Claussner, J.; Exner, J.; Feig, M.; Findeisen, S.; Hennig, C.; Kvashnina, K. O.; Naudet, D.; Prieur, D.; Rossberg, A.; Schmidt, M.; Qiu, C.; Colomp, P.; Cohen, C.; Dettona, E.; Dyadkin, V.; Stumpf, T. ROBL-II at ESRF: A Synchrotron Toolbox for Actinide Research. *J. Synchrotron Radiat.* **2021**, *28* (July 2020), 333–349. https://doi.org/10.1107/S1600577520014265.

(6) Mayordomo, N.; Rodriguez, D. M.; Schild, D.; Molodtsov, K.; Johnstone V, E.; Huebner, R.; Azzam, S. S. A.; Brendler, V.; Mueller, K. Technetium Retention by Gamma Alumina Nanoparticles and the Effect of Sorbed Fe2+. *J. Hazard. Mater.* **2020**, *388*. https://doi.org/10.1016/j.jhazmat.2020.122066.

(7) Rodriguez, D. M.; Mayordomo, N.; Scheinost, A. C.; Schild, D.; Brendler, V.; Mueller, K.; Stumpf, T. New Insights into Tc-99(VII) Removal by Pyrite: A Spectroscopic Approach. *Environ. Sci. Technol.* **2020**, *54* (5), 2678–2687. https://doi.org/10.1021/acs.est.9b05341.

(8) Fleet, M. E. The Structure of Magnetite. *Acta Crystallogr. Sect. B Struct. Crystallogr. Cryst. Chem.* **1981**, *37* (4), 917–920. https://doi.org/10.1107/s0567740881004597.

(9) Oliveira, A. F.; Kuc, A.; Heine, T.; Abram, U.; Scheinost, A. C. Shedding Light on the Enigmatic TcO2 · XH2O Structure with Density Functional Theory and EXAFS Spectroscopy**. *Chem. – A Eur. J.* **2022**, e202202235. https://doi.org/https://doi.org/10.1002/chem.202202235.

(10) Roßberg, A.; Reich, T.; Bernhard, G. Complexation of Uranium(VI) with Protocatechuic Acid-Application of Iterative Transformation Factor Analysis to EXAFS Spectroscopy. *Anal. Bioanal. Chem.* **2003**, *376* (5), 631–638. https://doi.org/10.1007/s00216-003-1963-5.

(11) Rüger, R.; Franchini, M.; Trnka, T.; Yakovlev, A.; van Lenthe, E.; Philipsen, P.; van Vuren, T.; Klumpers, B.; Soini, T. AMS 2022.1, SCM. Theoretical Chemistry, Vrije Universiteit: Amsterdam, The Netherlands 2022. http://www.scm.com.

(12) Te Velde, G.; Baerends, E. J. Precise Density-Functional Method for Periodic Structures. *Phys. Rev. B* **1991**, *44* (15), 7888–7903. https://doi.org/10.1103/PhysRevB.44.7888.

(13) Perdew, J. P.; Burke, K.; Ernzerhof, M. Generalized Gradient Approximation Made Simple. *Phys. Rev. Lett.* **1996**, *77* (18), 3865–3868. https://doi.org/10.1103/PhysRevLett.77.3865.

(14) Philipsen, P.; van Lenthe, E.; Snijders, J.; Baerends, E. Relativistic Calculations on the Adsorption of CO on the (111) Surfaces of Ni, Pd, and Pt within the Zeroth-Order Regular Approximation. *Phys. Rev. B - Condens. Matter Mater. Phys.* **1997**, *56* (20), 13556–13562. https://doi.org/10.1103/PhysRevB.56.13556.

(15) Jain, A.; Ong, S. P.; Hautier, G.; Chen, W.; Richards, W. D.; Dacek, S.; Cholia, S.; Gunter, D.; Skinner, D.; Ceder, G.; Persson, K. A. Commentary: The Materials Project: A Materials Genome Approach to Accelerating Materials Innovation. *APL Mater.* **2013**, *1* (1). https://doi.org/10.1063/1.4812323.

(16) Ong, S. P.; Richards, W. D.; Jain, A.; Hautier, G.; Kocher, M.; Cholia, S.; Gunter, D.; Chevrier, V. L.; Persson, K. A.; Ceder, G. Python Materials Genomics (Pymatgen): A Robust, Open-Source Python Library for Materials Analysis. *Comput. Mater. Sci.* **2013**, *68*, 314–319. https://doi.org/10.1016/j.commatsci.2012.10.028.

(17) Bianchetti, E.; Oliveira, A. F.; Scheinost, A. C.; Di Valentin, C.; Seifert, G. Chemistry of the Interaction and Retention of TcVII and TcIV Species at the Fe3O4(001) Surface. *J. Phys. Chem. C* **2023**, *4* (001). https://doi.org/10.1021/acs.jpcc.3c00688.

(18) Seah, M. P.; Gilmore, I. S.; Beamson, G. XPS%: Binding Energy Calibration of Electron Spectrometers 5ÈRe-Evaluation of the Reference Energies. **1998**, *649* (December 1997), 642–649.

(19) Huber, F.; Schild, D.; Vitova, T.; Rothe, J.; Kirsch, R.; Schäfer, T. U(VI) Removal Kinetics in

Presence of Synthetic Magnetite Nanoparticles. *Geochim. Cosmochim. Acta* **2012**, *96*, 154–173. https://doi.org/10.1016/j.gca.2012.07.019.

(20) Singh, M.; Ulbrich, P.; Prokopec, V.; Svoboda, P.; Šantavá, E.; Štěpánek, F. Vapour Phase Approach for Iron Oxide Nanoparticle Synthesis from Solid Precursors. *J. Solid State Chem.* **2013**, *200* (April), 150–156. https://doi.org/10.1016/j.jssc.2013.01.037.
